# Differential T cell response against BK virus regulatory and structural antigens: A viral dynamics modelling approach

**DOI:** 10.1371/journal.pcbi.1005998

**Published:** 2018-05-10

**Authors:** Arturo Blazquez-Navarro, Thomas Schachtner, Ulrik Stervbo, Anett Sefrin, Maik Stein, Timm H. Westhoff, Petra Reinke, Edda Klipp, Nina Babel, Avidan U. Neumann, Michal Or-Guil

**Affiliations:** 1 Berlin-Brandenburg Center for Regenerative Therapies (BCRT), Charité-Universitätsmedizin, Berlin, Germany; 2 Systems Immunology Lab, Department of Biology, Humboldt-Universität zu Berlin, Berlin, Germany; 3 Department of Nephrology and Internal Intensive Care, Charité-Universitätsmedizin, Berlin, Germany; 4 Medical Clinic I, Marien Hospital Herne, Ruhr University Bochum, Herne, Germany; 5 Theoretical Biophysics Group, Department of Biology, Humboldt-Universität zu Berlin, Berlin, Germany; 6 Institute of Environmental Medicine, UNIKA-T, Helmholtz Zentrum München, Augsburg, Germany; 7 Institute of Computational Biology, Helmholtz Zentrum München, Munich, Germany; Imperial College London, UNITED KINGDOM

## Abstract

BK virus (BKV) associated nephropathy affects 1–10% of kidney transplant recipients, leading to graft failure in about 50% of cases. Immune responses against different BKV antigens have been shown to have a prognostic value for disease development. Data currently suggest that the structural antigens and regulatory antigens of BKV might each trigger a different mode of action of the immune response. To study the influence of different modes of action of the cellular immune response on BKV clearance dynamics, we have analysed the kinetics of BKV plasma load and anti-BKV T cell response (Elispot) in six patients with BKV associated nephropathy using ODE modelling. The results show that only a small number of hypotheses on the mode of action are compatible with the empirical data. The hypothesis with the highest empirical support is that structural antigens trigger blocking of virus production from infected cells, whereas regulatory antigens trigger an acceleration of death of infected cells. These differential modes of action could be important for our understanding of BKV resolution, as according to the hypothesis, only regulatory antigens would trigger a fast and continuous clearance of the viral load. Other hypotheses showed a lower degree of empirical support, but could potentially explain the clearing mechanisms of individual patients. Our results highlight the heterogeneity of the dynamics, including the delay between immune response against structural versus regulatory antigens, and its relevance for BKV clearance. Our modelling approach is the first that studies the process of BKV clearance by bringing together viral and immune kinetics and can provide a framework for personalised hypotheses generation on the interrelations between cellular immunity and viral dynamics.

## Author summary

BK virus (BKV) is the cause of a kidney disease affecting 1–10% of kidney transplant recipients, which leads to transplantation failure in about 50% of the cases. This disease is not well understood, but there are indications that markers of the immune response against BKV can be used to predict the outcome. Since the immune response can act through different modes of action, we have studied the dynamics between immune response and virus to determine which modes of action play an important role in the fight against BKV. We have analysed immune and viral kinetics in six kidney transplantation patients and developed a mathematical model to integrate the data and better understand the interactions between virus and immune response to different BKV antigens. Our results allow for discarding the majority of action modes hypotheses. The most supported hypothesis is: structural proteins trigger the blocking of virus production by infected cells, whereas non-structural proteins trigger the acceleration of infected cells death. This difference could be central for disease outcome, as under this hypothesis only the latter would trigger a fast and continuous BKV clearance.

## Introduction

In the last years, BK virus-associated nephropathy (BKVN) has become the most challenging infectious cause of renal graft dysfunction in kidney transplant, leading to graft failure in over 50% of cases [1,2]. The rise in BKVN incidence has been attributed, at least to some degree, to the increased potency of immunosuppressive drugs [3,4]. Given the absence of specific antiviral treatments, BKVN is handled by changing the immunosuppressive regimes of the patients, enabling the development of a specific antiviral immune response [3–5]. Diagnosis of BKVN is performed through renal biopsy [3,6–8] as progression of the illness occurs without clinical signs, except for an increase in serum creatinine concentrations [1]. In the absence of medical intervention, BKVN can cause extensive fibrosis and tubular atrophy in the allograft, leading to transplant loss [1,3,7]. This progression is accompanied by a high BK virus (BKV) plasma load. Therefore, screening of plasma BKV viral load is currently recommended for the monitoring of BKVN [8,9].

BKV is a non-enveloped virus with an icosahedral capsid and a small circular double-stranded DNA genome (~5kb), which encodes for the early regulatory proteins: small tumor antigen (st) and large tumor antigen (LT) (here collectively referred to as sLT antigens), the late structural viral proteins 1–3 (VP1, VP2 and VP3) (here referred to as VP antigens) and the agnoprotein [3,10]. Latent BKV infection is very common among the healthy population, with a prevalence above 80% [3,11–13].

In spite of a high frequency of self-limited BKV reactivation in kidney transplant recipients [12,14,15], only 1–10% [2] of transplant recipients do actually develop BKVN. To determine the factors leading to BKVN, much emphasis has been placed on the immune reaction against BKV antigens. sLT and VP antigens (but not the agnoprotein) have been demonstrated to elicit a T cell response, as we previously showed in our studies [16–18]. Our data suggest that cellular immune reaction has a prognostic value for BKVN evolution [16]. However, T cell response can act through a number of mechanisms—killing of infected cells, blocking virus production or infection, among others—which should have different impacts on viremia control. Although our data [16] suggest that VP and sLT antigens trigger substantially different immune responses, the experimental data alone do not allow to determine the relation between antigens, immune mechanisms and clearance. Sophisticated instruments, such as mathematical models tailored for data analysis of this particular question, are required to formalise and analyse whether different antigens trigger different immune mechanisms and what these modes of action are.

The most widely used method for modelling viral dynamics is ordinary differential equations (ODE). It has, for instance, helped elucidate the dynamics of HIV-1, hepatitis and opportunistic viruses in transplant recipients [19]. It has also been used for the study of BKV, simulating the dynamics of viral production, predicting cytopathic effects of the virus and explaining the interactions between viral reactivation in tubular epithelial cells, urothelial cells, viremia and viruria [20,21]. However, to our knowledge, no model exists that incorporates the activation of the immune response with viral clearance dynamics.

Therefore, in this study we have retrospectively analysed the data of BKV plasma load kinetics and T cell responses against BKV antigens in six patients with biopsy-proven BKVN [16]. The objective of the analysis was to determine the dominant modes of action of the observed immune responses. For this, a tailor-made ODE model was generated, allowing for the formalisation of different hypotheses on the dominant modes of action of the immune response against BKVN.

To accomplish our goal, we pursued the following strategy: Firstly, we obtained a continuous curve that fits the time course of the T cell response data (Elispot) for each patient and antigen. Secondly, we designed an ODE model for the viral load clearance dynamics dependent on the T cell response curves. This model uses the former curves as input and simulates the dynamics of three variables: number of healthy cells, number of infected cells and BKV viral load. It incorporates three mechanisms of the immune system in viral clearance, allowing for the simulation of nine different hypotheses about dominant modes of action. Lastly, we evaluated all hypotheses for their capacity to reproduce the viral clearance data. Our results allowed for the discarding of most hypotheses and suggested that the anti-VP response induces the blocking of virus production while anti-sLT responses induces killing of infected cells. This difference in modes of action could be central for disease outcome, since only the sLT responses would trigger a fast and continuous BKV clearance under this hypothesis. These results could therefore have implications in the development of new immunotherapies against BKVN.

## Results

### Patient characteristics and clinical data

The study involved six renal transplant patients analysed in our previous study [16]. These six patients (called Patient A to F in the following) received renal transplants between 12/2004 and 05/2009 and developed severe BKV reactivation in follow-up. The patients were monitored for BKV viral load by quantitative polymerase chain reaction (qPCR). Cellular adaptive immune response against the BKV antigens (VP1, VP2, VP3, st and LT) was monitored by Interferon gamma (IFN-γ) Enzyme-Linked ImmunoSpot (Elispot), measured in spot forming units (SFU) per 10^6^ peripheral blood mononuclear cells (PBMC). Elispot read-outs are known to accurately quantify antigen-specific T cell responses for BKV [22].

**Fig 1:**
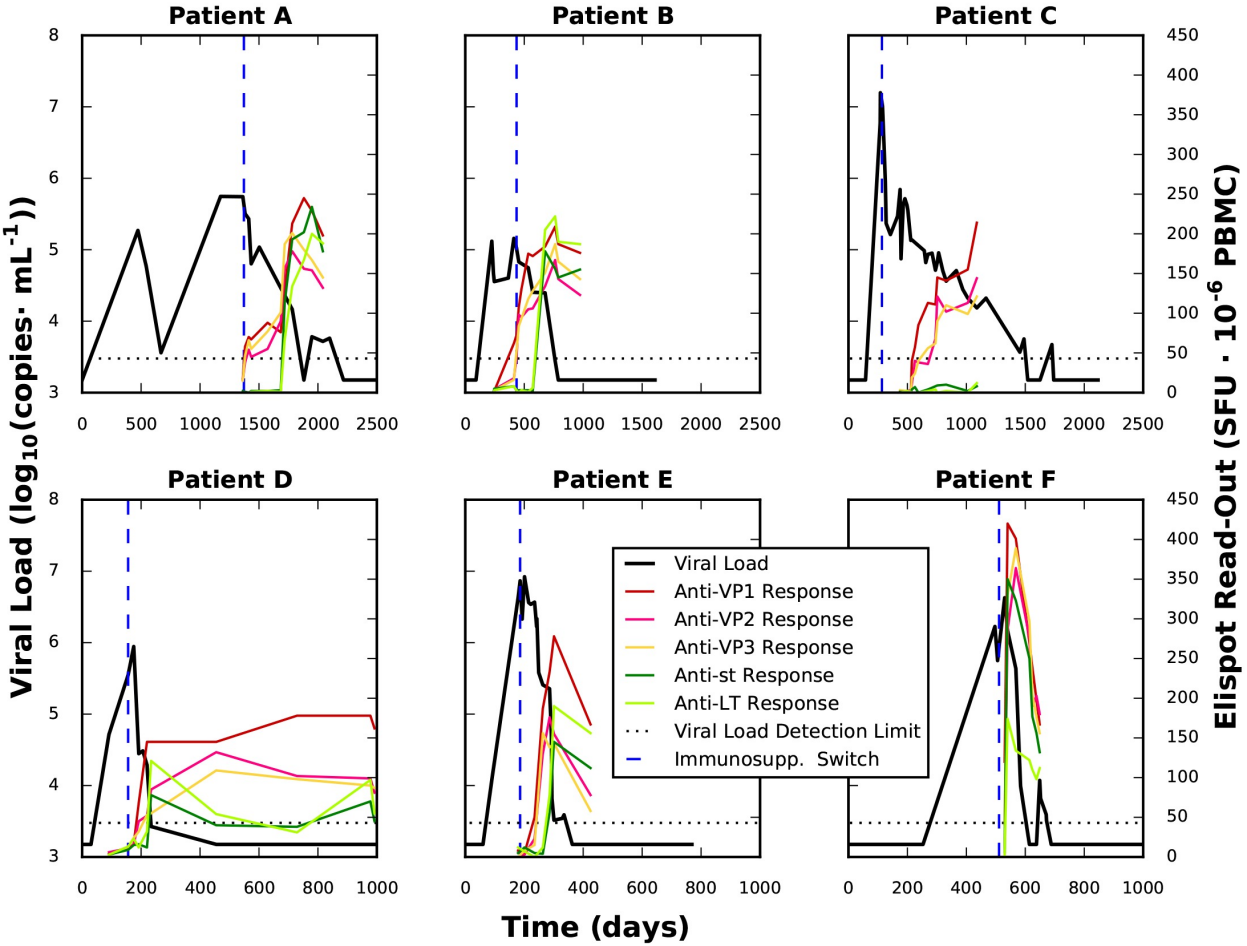
Viral load and immune response data of the patients. For each patient, the time course of viral load (black) and the Elispot read-out for each immunogenic BKV antigen (coloured) are plotted. The change of immunosuppressant therapy is marked as a dashed blue line. This change in immunosuppressant therapy is known to foster the development of an immune response against BKV. On the upper row the patients that had not cleared within 700 days after transplantation are shown, while those that achieved clearance in a shorter time appear in the lower row. Please note the difference of time scales between the rows.

All patients had biopsy-proven BKVN and were initially treated with a tacrolimus-based immunosuppressive regimen. Tacrolimus is a calcineurin inhibitor. It inhibits T cell activation but does not have cell-depleting effects [23]. It is associated with significantly higher incidence of BKVN compared to cyclosporine A, a less potent calcineurin inhibitor [24]. Upon BKV reactivation and diagnosis of BKVN, tacrolimus was replaced by cyclosporine A. This immunosuppressant switch is a commonly used protocol against BKVN, as cyclosporine A is known to allow the onset of a T cell response against BKV [16,25]. Patients were monitored for BKV viral load during the complete evolution of the illness. The immune response was measured at the latest from the point of immunosuppressant switch until BKV clearance (Fig 1).

### Description of viral load and Elispot experimental data

We observed a considerable diversity in the times needed to reach viremia clearance for each patient, ranging from 117 days after viremia onset for Patient F to 1744 days (~4 years) for Patient A. However, some common patterns could be observed. The immune response came generally in two waves, the first with an anti-VP immune response (red, pink and yellow lines in Fig 1) and the second, targeted against sLT antigens (light and dark green). Importantly, the immune response against VP was triggered for all but patient C within a relatively short span of time (< 70 days) after immunosuppressant switch. On the other hand, immune response against the sLT antigens was observed in only five patients. Again patient C did not show any immune response against either sLT antigen. Based on the delay between the VP and the sLT immune responses, patients could be grouped in two categories: Patients D, E and F showed a short delay of approximately 30 days, while patients A and B showed a much longer delay of over 180 days.

The triggering of cellular immune responses against the BKV antigens occurred after the immunosuppressant switch. This immune response led to a progressive decrease of viral load until viral clearance was achieved. This decreasing phase took place for hundreds of days on most cases. In the five patients showing an anti-sLT immune response, the emergence of this response was tied to a substantially faster viral load decrease. This strongly suggests that the kind of immune response triggered by the sLT antigens is inherently different from the one triggered by VP antigens.

### Fitting of a model of the immune response against BKV to obtain continuous curves describing the T cell response

With the goal of using the immune response data as an input for the viral load clearance dynamics model, we developed a simple curve based on one or more logistic functions to describe the experimentally observed T cell response. The use of logistic functions to describe T cell dynamics of antigen specific populations was chosen due to their simplicity and capacity to describe saturation-limited growth processes [26–28]. The model for one logistic function is

$$\begin{array}{ll} \frac{\text{d}}{\text{d}t}anti_{a}\left(t\right)= & \{\begin{array}{ll} 0, & \text{for}\;0\leq t\leq t_{a}\\ r_{a}\cdot anti_{a}\left(t\right)\cdot \left(1-\frac{anti_{a}\left(t\right)}{max_{anti_{a}}\cdot \left(1-dec_{a}\cdot t\right)}\right), & \text{for}\;t>t_{a} \end{array} \end{array}$$ (1)

*anti*_*a*_*(t)* is the T cell response for an antigen, where *a* represents the antigen that elicits the response. For the definition of parameters see Table 1. We chose the activation time *t*_*a*_ as a free parameter because the T cell response may start at different points in time for every antigen. As it is possible that an immune response presents multiple boosting episodes, we considered the possibility that at a second time point t_a2_ the parameters of the curve are replaced by a second set of parameters. We fitted this function to the BKV specific immune response against each of the five antigens (VP1, VP2, VP3, st and LT).

**Table 1:** Immune function curve parameters.

Name	Meaning	Unit
*t*_*a*_	Activation time of immune response	Days
*r*_*a*_	Immune response growth rate	Days^-1^
*max*_*antia*_	Maximum immune response	SFU · 10^−6^ PBMC
*dec*_*a*_	Maximum response decay rate	Days^-1^

Definition of the parameters of the immune function curve (Eq 1)

**Fig 2:**
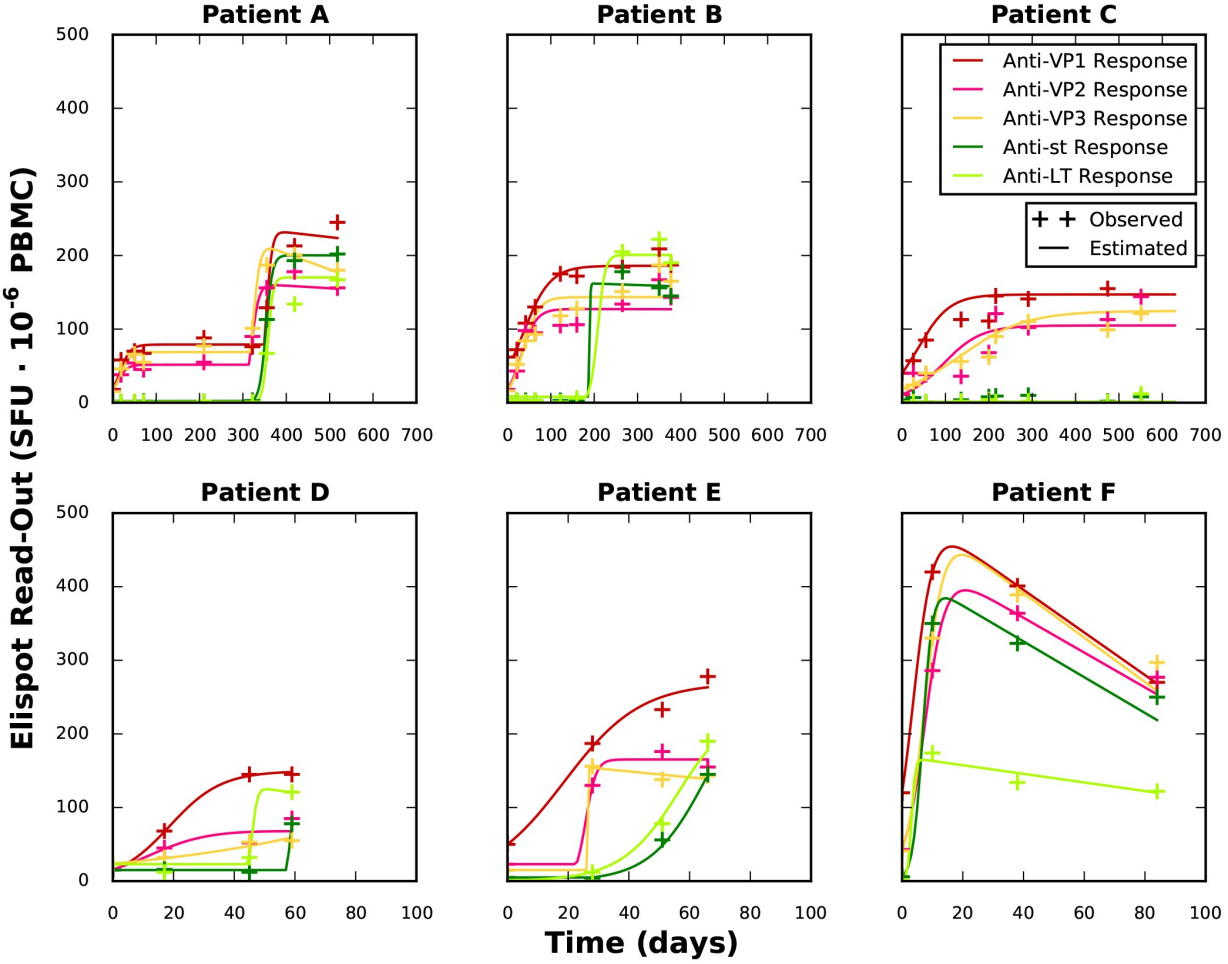
Fitting of immune response data. The calculated values for the immune response (lines) are plotted against the observed values (plus sign). Note the difference of time scales between the rows.

*t* = 0 was defined at a day for which there are both Elispot and viral load data and the viral load is maximum compared to all later measurements. This was defined as follows: Patient A, day 1363 after transplantation; B, day 412; C, day 538; D, day 175; E, day 235; and F, day 530. Simulations were performed until the time point viral load becomes undetectable or there are no further Elispot measurements. This time point was chosen because we aim to model only the clearance process. The objective function used for the fitting takes the form of vertical least-squares such that

$$f\;=\;\frac{\sum_{t\;=\;1}^{N}\sum_{a\;=\;1}^{A}{\left({log}_{10}(\overset{-}{y}(t,a))-{log}_{10}(y(t,a,p))\right)}^{2}}{N}$$ (2)

where $\overset{-}{y}(t,a)\;$ is the experimental value of the Elispot read-out at time *t* for antigen *a*. *y(t*,*a*,*p)* is the calculated Elispot read-out for a given parameter set *p*. *N* is the total number of measurements and *A* is the number of screened antigens. The results of the parameter estimation are shown in S1 Table and Fig 2. As depicted in Fig 2, Eq 1 was sufficient to reproduce the immune response time courses of all six patients. For the immune response to the structural antigens of Patient A, a time point t_a2_ with a second parameter set was employed to achieve a minimum value for the objective function of (4.16·10^−2^), instead of the minimum achieved for only one parameter set (2.24·10^−1^) (see S1 Fig).

In order to study the differences in the mechanisms of the immune responses against structural (VP1, VP2, VP3) and regulatory (st, LT) antigens, the results of the fitting were summarised in a VP function and a sLT function. These functions are employed in the model of BKV viral load clearance as an input, to model the influence of each immune response against BKV.

$$VP(t)\;=\;max({anti}_{VP1}(t),{anti}_{VP2}(t),{anti}_{VP3}(t))-1$$

$$sLT(t)\;=\;max({anti}_{st}(t),{anti}_{LT}(t))-1$$ (3)

The maximum value is taken under the assumption that the effects of the antigens are not additive, but that there is some degree of saturation. The functions are subtracted by one unit because 1 is the baseline value of the logistic curve *anti*_*a*_*(t)*.

### Model of BKV viral load clearance in dependence on immune response time course

The evolution of BKV viral load clearance was described using a modified version of a basic model of viral dynamics [29], such that

$$\frac{d}{dt}C(t)\;=\;g\cdot C(t)\cdot (1-\frac{C(t)+I(t)}{{max}_{c}\;})-d\cdot C(t)-\beta \cdot C(t)\cdot V(t)\cdot (1-\nu (t))$$

$$\frac{d}{dt}I(t)\;=\;\beta \cdot C(t)\cdot V(t)\cdot (1-\nu (t))-d\cdot k\cdot I(t)\cdot (1+m\cdot \mu (t))$$

$$\frac{d}{dt}V(t)\;=\;p\cdot I(t)\cdot (1-\epsilon (t))-c\cdot V(t)$$ (4)

**Fig 3:**
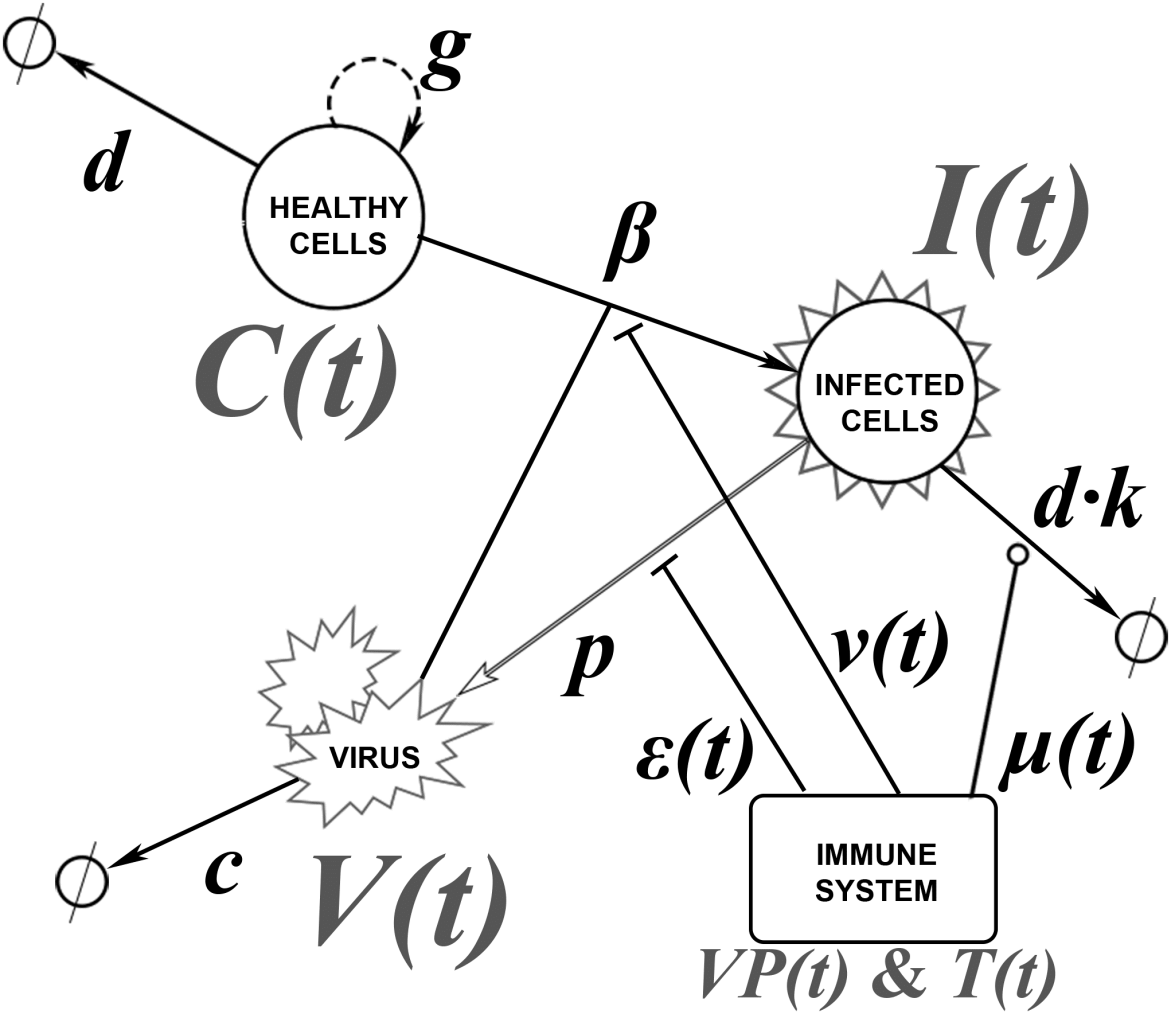
Schematic representation of the ODE model. Healthy cells produce other healthy cells (rate proportional to *g*) and die at rate *d*. The virus triggers the conversion of healthy cells into infected cells (rate *β*). Infected cells die at rate *d·k* and produce the virus at rate *p*, which is cleared at rate *c*. The immune system can intervene through three different mechanisms: blocking virus production (*ε(t)*), enhancing infected cell death (*μ(t)*) and blocking infection (*ν(t)*).

This model contains three variables: number of healthy cells (*C*), number of infected cells (*I*) and BKV viral load in copies · mL^-1^ (*V*). Healthy cells proliferate at a rate proportional to *g*; this rate is limited by *max*_*c*_, which represents total number of cells (including both healthy and infected). Healthy cells die at a rate *d* and are infected in presence of virus at a rate *β*. Infected cells die at a rate *d · k*, where *k* is virus-associated cytopathicity. Viruses are produced by the infected cells at a rate *p* and get cleared by the excretory system at a rate *c*. For a schematic representation of the model, see Fig 3. For a further definition of the parameters, see Table 2.

**Table 2:** Viral load clearance model parameters.

Name	Meaning	Unit
*g*	Self-regeneration of healthy cells rate	Days^-1^
*max*_*c*_	Maximum number of total cells	Cells
*d*	Cell death independent of viral cytotoxicity rate	Days^-1^
*β*	Cell infection rate	Copies^-1^ · mL · days^-1^
*k*	Viral cytopathicity factor	Unitless
*p*	Virus production rate	Copies · mL^-1^ · cells^-1^ · days ^-1^
*c*	Virus clearing rate	Days^-1^
*m*	Maximum value of accelerated killing with *μ*(*t*)	Unitless

Definition of the parameters of the viral load clearance model (Eq 4)

The three model variables (*C*, *I* and *V*) depend on the T cell response curves as defined in previous section. To study the mode of action of T cell responses, we consider that T cells can act via three mechanisms: (1) virus production blockage (described by function *ε*(*t*)), (2) killing of the infected cells (described by function *μ*(*t*)) and (3) infection blockage (described by function *υ(t)*). *ε*(*t*), *μ*(*t*) and *υ(t)* take the form of the sum of Hill functions, a standard form for describing a saturating function, with a maximum value of 1, such that

$$\epsilon (t)\;=\;{max}_{\epsilon }\cdot \frac{{VP(t)}^{{hill}_{\epsilon }}}{{\theta_{\epsilon }^{{hill}_{\epsilon }}+VP(t)}^{{hill}_{\epsilon }}}+{max}_{Ε}\cdot \frac{{sLT(t)}^{{hill}_{Ε}}}{{\theta_{Ε}^{{hill}_{Ε}}+sLT(t)}^{{hill}_{Ε}}}$$

$$\mu (t)\;=\;{max}_{\mu }\cdot \frac{{VP(t)}^{{hill}_{\mu }}}{{\theta_{\mu }^{{hill}_{\mu }}+VP(t)}^{{hill}_{\mu }}}+{max}_{Μ}\cdot \frac{{sLT(t)}^{{hill}_{Μ}}}{{\theta_{Μ}^{{hill}_{Μ}}+sLT(t)}^{{hill}_{Μ}}}$$

$$\nu (t)\;=\;{max}_{\nu }\cdot \frac{{VP(t)}^{{hill}_{\nu }}}{{\theta_{\nu }^{{hill}_{\nu }}+VP(t)}^{{hill}_{\nu }}}+{max}_{Ν}\cdot \frac{{sLT(t)}^{{hill}_{Ν}}}{{\theta_{Ν}^{{hill}_{Ν}}+sLT(t)}^{{hill}_{Ν}}}$$

$${max}_{\epsilon }+{max}_{Ε}\leq 1$$

$${max}_{\mu }+{max}_{Μ}\leq 1$$

$${max}_{\nu }+{max}_{Ν}\leq 1$$ (5)

where *ε*(*t*), *μ*(*t*) and *υ(t)* depend on the *VP(t)* and the *sLT(t)* immune responses, as defined in Eq 3.

### Hypotheses on immune modes of action on the BKV viral load clearance model

The objective of our work is to find the dominant modes of action responsible for viral load clearance. Therefore, we assume for the model that each one of the two immune responses (*VP(t)* and *sLT(t*)) acts through only one mode of action, either *ε*(*t*), *μ*(*t*) or *υ(t)* (Eq 5). As these are three modes of action and two antigen-specific responses, nine different hypotheses on the relationship between dominant modes of action and immune response are possible. These nine hypotheses are referenced here following this convention: For example, the hypothesis that anti-VP triggers a *μ(t)* response (accelerated killing) and anti-sLT triggers a *υ(t)* response (infection blockage) is named VPμ-sLTυ hypothesis. For the definition and description of all nine hypotheses, see S2 Table.

### Testing of hypotheses for dominant modes of action of the immune system in BKV clearance

To evaluate the feasibility of the hypotheses for dominant modes of action of the immune system, the model was fitted against the BKV clearance data for all nine hypotheses. The parameters *c*, *g*, *d*, *β* and *p* were estimated based on previous publications. Parameter *k* was estimated for each hypothesis based on one particular patient, while the remainder of the parameters were estimated individually for each patient and hypothesis.

The rate constant *c* for virus clearance was fixed to the value calculated by Funk *et al*. [30]. In the case of *g*, which is the maximum replication capacity for *C(t) + I(t) << max*_*c*_, cell culture results show a maximum duplication rate of approximately one day for renal foetal kidney cells [31]. Therefore, for the sake of simplicity we assigned a value of 1 days^-1^ for *g*. For the cell death rate of healthy cells *d*, a value of 0.01 days^-1^ was used on a model of similar structure for Hepatitis C virus [32] and it was deemed to be reasonable estimation here. The value of the virus production rate *p* was calculated in the same model to be 100 copies · mL^-1^ · cells^-1^ · days^-1^ [32]. Given that BKV is a less aggressive infection, we deemed it reasonable to assume a value of 15 copies · mL^-1^· cells^-1^ · days^-1^. This has the property that, for *I(t) = V(t)* and no immune reaction, the viral load is in a steady state. Likewise, as the cell infection rate *β* for the Hepatitis C virus was estimated to be 3·10^−7^ copies^-1^ · mL · days^-1^ [32], a value of 3·10^−8^ copies^-1^ · mL · days^-1^ for BKV was assumed. Patient C had the slowest progression of viral clearance, which suggests that immune cytotoxic effects were relatively low. Therefore, we estimated the viral cythopathic factor (*k*) for all patients using data obtained from Patient C. The model as defined by Eqs 3–5 was fitted for all nine hypotheses (S2 Table) with the objective function

$$f\;=\;\frac{\sum_{t\;=\;1}^{N}{\left({log}_{10}(\overset{-}{y}(t))-{log}_{10}(y(t,p))\right)}^{2}}{N}$$ (6)

which takes the form of vertical least-squares. *N* is the total number of measurements, $\overset{-}{y}(t)$ is the viral load at the time *t*, *y(t*, *p)* is the simulated viral load for a parameter set *p* and time *t*. The initial conditions for all cases were

For *t* = 0:

$$C(0)\;=\;{max}_{c}-\frac{c}{p}\cdot V(0)$$

$$I(0)\;=\;\frac{c}{p}\cdot V(0)$$

V(0)=V(0) (7)

so that, at time *t* = 0 and no immune response, viral load is in steady state. *V(0)* is defined as the observed viral load at *t* = 0. *t* = 0 was defined as above. The results obtained for the fittings, as well as the model selection criterion (see Materials and methods) for each hypothesis and patient, are shown in Table 3.

**Table 3:** Results of the model fitting for the hypotheses on dominant immune modes of action.

Patient	Measurement	VPε-sLTε	VPε-sLTμ	VPε-sLTν	VPμ-sLTε	VPμ-sLTμ	VPμ-sLTν	VPν-sLTε	VPν-sLTμ	VPν-sLTν
**Number parameters**		7	6	5	6	7	6	5	6	7
**A**	***f***	0.11957	**0.03090**	0.06613	0.05490	0.04409	0.06091	0.14584	0.05053	1.88160
	**ΔBIC**	11.4180	**0.0000**	3.3800	4.0233	4.4336	4.7510	8.9165	3.4425	30.7098
**B**	***f***	0.06843	**0.01120**	0.06046	0.03153	0.02190	0.02233	0.06159	0.01994	0.06227
	**ΔBIC**	14.6151	**0.0000**	9.8562	7.2449	6.6399	4.8297	9.9857	4.0387	13.9550
**C**	***f***	0.01280	0.01030	0.01230	0.01070	0.01070	0.01070	0.01050	0.01050	**0.01020**
	**ΔBIC**	5.6813	**0.0000**	2.48438	**0.5334**	3.1725	**0.5334**	**0.2692**	**0.2692**	2.5025
**D**	***f***	**0.00005**	0.00044	0.10923	0.01080	0.18563	0.15925	0.01870	0.13048	2.33900
	**ΔBIC**	**0.0000**	7.2157	27.9327	20.0636	32.8264	30.8273	20.8732	30.0301	42.9616
**E**	***f***	0.17314	0.05591	0.25664	**0.05083**	0.08718	0.11018	3.03501	0.28754	2.40041
	**ΔBIC**	11.8850	**0.7632**	10.8748	**0.0000**	6.3957	6.1895	30.6372	13.8637	32.9195
**F**	***f***	1.25703	0.15598	1.31113	0.24925	0.21315	**0.12060**	1.31455	0.12063	4.25438
	**ΔBIC**	10.7624	**1.0289**	8.1584	2.9039	3.6644	**0.0000**	8.1688	**0.0008**	15.6392
***f***_**SUM**_		1.63101	**0.26472**	1.81587	0.40800	0.56264	0.48397	4.58619	0.61961	10.94786
**Median ΔBIC**		11.0902	**0.3816**	9.0073	3.4636	5.4146	4.7903	9.4511	3.7406	23.1745

The results for the objective function *f* (Eq 6) and ΔBIC (Eqs 8 and 9) are shown for each one of the hypotheses and patients. The sum of the objective functions over all patients is shown as *f*_SUM_. In bold are highlighted: The lowest per patient values for *f*, as well as the scores of ΔBIC within the range of substantial empirical support (<2). The definitions of the hypotheses are shown in S2 Table. Detailed results of the model selection criteria are shown in S3 Table. S2 Fig shows the results of the fittings for each hypothesis, compared to the best-performing hypothesis.

The results in Table 3 were interpreted to discard hypotheses based on the ΔBIC score and the value of the objective function. Accordingly, there is good empirical support to generally discard hypotheses VPν-sLTν and VPν-sLTε as probable mechanisms for viral clearance. Hypotheses VPμ-sLTν, VPε-sLTν, VPε-sLTε, VPμ-sLTμ and VPν-sLTμ can only be considered as possible mechanisms for individual patients but not for the entire patient cohort. Hypothesis VPμ-sLTε cannot be discarded but does not show the highest degree of empirical support.

The hypothesis VPε-sLTμ has the lowest median ΔBIC and thus the highest empirical support. For five out of six patients, this hypothesis was within the range of substantial empirical support (ΔBIC <2) [33], while no other hypothesis had comparable support for more than two patients. This hypothesis associates an anti-VP response with virus production blockage and an anti-sLT response with accelerated killing of infected cells. The hypothesis VPε-sLTμ is shown compared to the other alternative hypotheses in S2 Fig.

**Fig 4:**
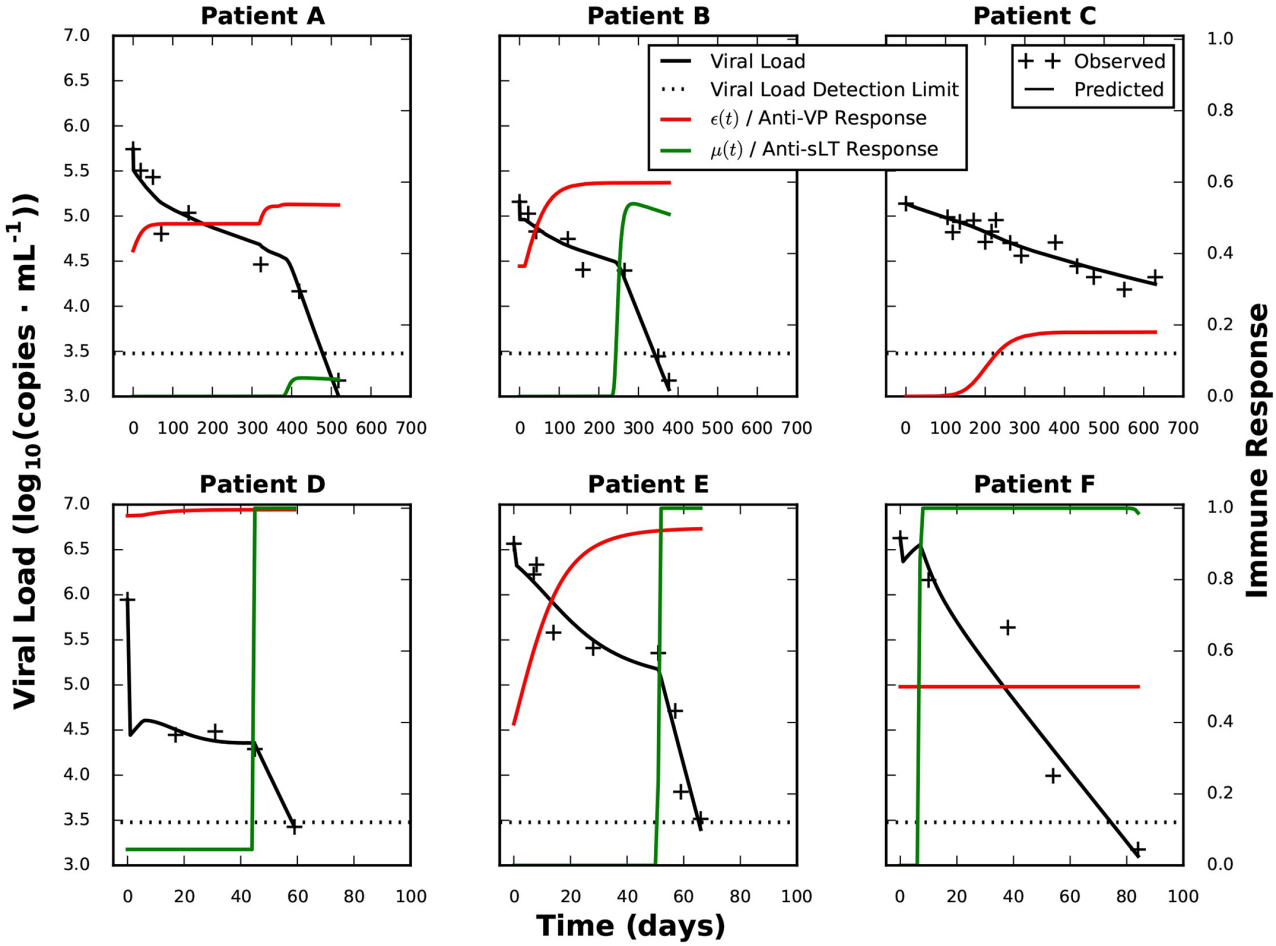
Modelled time course of BKV viral load clearance for hypothesis VPε-sLTμ. The results of the model (Eqs 3–5) under hypothesis VPε-sLTμ (S2 Table) using the parameters in Table 4 are plotted: viral load (*V*(t)) is shown as a black line, the immune responses virus production blockage (*ε*(t)) and accelerated killing of infected cells (*μ*(t)) are shown in green and red, respectively. Observed viral load values are shown as black plus signs. Please note the difference of time scales between the rows.

Results of the parameter estimation, confidence intervals and the objective function for the VPε-sLTμ hypothesis are shown in Table 4. The fitted model for each patient is shown on Fig 4.

**Table 4:** Parameter for the viral load clearance model under hypothesis VPε-sLTμ.

		Patients					
Parameter	Type	A	B	C	D	E	F
*g*	Fixed value	1.00					
*d*	Fixed value	1.00·10^−2^					
*p*	Fixed value	15.00					
*β*	Fixed value	3·10^−8^					
*c*	Fixed value	15.00					
*k*	Fixed value	1.02					
*max*_*c*_	Estimated value	5.52·10^5^	6.91·10^5^	3.39·10^5^	1.91·10^8^	3.78·10^6^	1.17·10^7^
	95% Confidence interval	[5.52·10^5^, 7.07·10^5^]	[5.44·10^5^, 1.46·10^6^]	[2.86·10^5^, 3.62·10^5^]	[1.43·10^8^, 2.64·10^8^]	[3.69·10^6^, 1.43·10^9^]	[4.25·10^6^, 5.53·10^7^]
*m*	Estimated value	48.3	4.27	-	15.8	25.9	24.9
	95% Confidence interval	[47.3, 55.9]	[4.21, 4.86]	-	[13.2, 18.5]	[16.4, 41.6]	[11.5, 86.5]
*hill*_*ε*_	Estimated value	2.00·10^−1^	8.59·10^−1^	1.12·10^2^	8.93·10^−1^	1.92	1.82·10^−9^
	95% Confidence interval	[1.85·10^−1^, 2.07·10^−1^]	[8.44·10^−1^, 8.61·10^−1^]	[1.07·10^2^, 1.15·10^2^]	[7.96·10^−1^, 9.05·10^−1^]	[1.00, 2.88]	[1.80·10^−61^, 4.45·10^−1^]
*θ*_*ε*_	Estimated value	1.08·10^2^	1.16·10^2^	1.48·10^2^	3.15·10^−1^	61.0	78.7
	95% Confidence interval	[1.04·10^2^, 1.37·10^2^]	[48.2, 1.45·10^2^]	[1.36·10^2^, 1.70·10^2^]	[9.02·10^−2^, 3.76·10^−1^]	[5.93, 89.4]	[3.07·10^−4^, 1.68·10^8^]
*hill*_*μ*_	Estimated value	1.30·10^2^	1.34·10^2^	-	98.6	1.36·10^2^	1.13·10^2^
	95% Confidence interval	[1.29·10^2^, 1.30·10^2^]	[1.28·10^2^, 1.34·10^2^]	-	[47.5, 1.49·10^2^]	[38.8, 1.74·10^2^]	[19.5, 1.49·10^2^]
*θ*_*μ*_	Estimated value	2.04·10^2^	2.00·10^2^	-	22.7	87.3	2.10·10^2^
	95% Confidence interval	[2.03·10^2^, 2.04·10^2^]	[1.99·10^2^, 2.00·10^2^]	-	[22.2, 29.2]	[50.0, 1.19·10^2^]	[1.37, 3.28·10^2^]
***f***	**Obj. Function**	**3.10·10**^**−2**^	**1.12·10**^**−2**^	**1.03·10**^**−2**^	**4.40·10**^**−3**^	**5.60·10**^**−2**^	**1.56·10**^**−1**^

Results of the fitting for the viral clearance model (Eqs 3–5) under hypothesis VPε-sLTμ (S2 Table) for all six patients. The last row indicates the value of the objective function (Eq 6).

In spite of the good results of the fitting, the estimated values of the parameters should be taken with caution. The results show heterogeneity between patients, especially *max*_*c*_, *hill*_*ε*_ and *θ*_*ε*_, with a range of around 3 orders of magnitude. This could be partly caused by parameter uncertainty, as supported by the 95% confidence intervals, which for some parameters range over 2 orders of magnitude. However, the variation of parameters between patients is larger than the confidence intervals for each patient, confirming that the high variation is not solely a product of parameter uncertainty. This is not surprising, as there is a very high degree of variation in the clearing time courses of the patients.

Note that the fitted parameters summarise complex biological processes, as opposed to reflecting fundamental mechanisms, rendering it difficult to interpret parameter variations. Nevertheless, fundamental biological variation between patients is conceivable. A clear case is patient F. This patient had a simultaneous activation of the anti-VP and anti-sLT immune response, and the extremely low estimate for *hill*_*ε*_ and very broad confidence intervals *hill*_*ε*_ and *θ*_*ε*_, suggest that the anti-sLT immune response through the *μ* mode of action could have a saturating effect over the anti-VP immune response. In fact, assuming only an anti-sLT response for patient F led to an increase of *f* of less than 5% in comparison to the original VPε-sLTμ, with substantially lower BIC values (see S3 Table). This result supports the possibility of a saturating anti-sLT response for this patient.

### Sensitivity analysis of the model

To analyse the impact of the chosen values for the fixed parameters *g*, *d*, *p*, *β* and *k* on the behaviour of the model, a sensitivity analysis was performed. The goal was to analyse whether the same quality of fitting and qualitative behaviour of the model can be achieved for different values of these parameters. The analysis was performed following the principle of one-factor-at-a-time. The value of a single parameter was modified over a span ranging from a factor 0.1 of the original value up to 10; for each new value of the parameter a fitting was performed to minimise the value of the objective function (Eq 6). The detailed results of the sensitivity analysis are shown in S4 Table, the results for the extreme values (factors 0.1. and 10) are plotted in S3 Fig.

Briefly, the results show that the model VPε-sLTμ can robustly simulate the viral clearance dynamics of the six patients and is not sensitive to variations of the fixed parameters: For the extreme values (factors 0.1 and 10), fittings with *f*_SUM_ < 0.4 were achieved in all cases. This is especially relevant when comparing the results with those for the mode of action hypotheses (Table 3), where the best alternative hypothesis had a *f*_SUM_ = 0.40800. Taken together, the results of the analysis reinforce the relevance of the hypothesis VPε-sLTμ, demonstrating that it is able to fit the viral dynamics better than the other hypotheses, even when modifying the fixed parameters across two orders of magnitude.

## Discussion

In this work we have created the first model that provides evidence of the dominant modes of action involved in the clearance of BKV. It is the first model that covers the process of BKV clearance harmonising the viral and immune dynamics and formalising different modes of action of the immune system and their influence on the viral dynamics. It incorporates the influence of the adaptive immune system on the clearance of BKV reactivation in a patient-to-patient basis by considering multiple antigens and immune reactions against the same viral infection and highlighting certain patterns of the process of immunological re-arming against BKV after immunosuppressant switch. Our results show that immune modes of action can be captured by acquisition of time series of blood markers not directly related to mechanistic observations. Taken together, our work can be used as a tool for personalised hypothesis generation and evaluation of the modes of action through which the immune system successfully fights against BKVN.

Our model suggests that for VP-specific cellular immune response, the dominant mode of action is reducing the rate of virus production, while the mode of action triggered by sLT-antigen specific T cells is an increased death rate of infected cells. This remarkable feature would be central for BKV clearance: the VP-triggered immune response would cause an initial drop in the viral load, leading to a plateau, where reduction of the viral load is slower than 0.5 log_10_(virus·mL^-1^) every 100 days. Only the acceleration of death of infected cells, triggered by the sLT antigens, would lead to a fast and continuous clearance of the viral load. It further suggests that in cases of simultaneous anti-VP and anti-sLT response, the latter response would play the central role in viral clearing.

This hypothesis, VPε-sLTμ, achieved substantial empirical support for five out of six patients, while none of the alternative hypotheses on dominant modes of action had substantial empirical support for more than two patients. Even though one alternative hypothesis could be used to fit the viral dynamics of the patients satisfactorily, the VPε-sLTμ hypothesis achieved the lowest total value for the objective function.

The suggested VP-triggered blockage of virus production can be linked mechanistically to the action of some cytokines, such as type I-interferons; while sLT-triggered accelerated killing can be associated with cytotoxic cells. This qualitatively different role of both antigen groups is in agreement with biological evidence provided by a previous flow cytometry-based study on VP1- and LT-specific CD4^+^ and CD8^+^ T-cells in patients with BKV reactivation [34]. In this work, VP1 elicited a significantly higher response in CD4^+^ T-cells than in CD8^+^ T-cells. In the case of the LT antigen, even though there was no significant difference between the magnitude of the CD4^+^ and the CD8^+^ T-cell responses, CD8^+^ cells were significantly more likely to respond against LT than VP1. The agreement between the hypothesis with the highest empirical support and the cited study highlights, in our opinion, the capabilities of using our model as an instrument for hypothesis generation on the physiological background of BKV clearance.

Interestingly, our model highlights a feature of heterogeneity among patients, the delay between anti-VP and anti-sLT immune response, as central for BKV dynamics, linking it to the previously presented division of patients into two groups—with a first group (upper row in Fig 4) clearing the infection after over 300 days and a second group (lower row) clearing the infection in around 100 days after immunosuppressant switch—in terms of an increased clearance speed associated with anti-sLT immune response. Our model highlights the close relationship between viral clearance and this delay, underscoring that anti-sLT specific T cells are needed for clearance. A delay between VP and sLT responses has been observed in two previous studies [16,34]. However, in spite of having been observed repeatedly, there is to our knowledge at present no discussion in the literature on this striking factor. Possible causes could be related to the different ways of VP and sLT antigen presentation or to the effects of immunosuppression. Based on the results of our model, we would welcome more profound experimental and theoretical research on the reasons underlying the delay.

Moreover, our results suggest that heterogeneity is not confined to the delay between immune responses but is a central feature of the BKV clearing dynamics: For certain individual patients, hypotheses other than VPε-sLTμ might be specifically suitable to explain their viral clearance dynamics. There is also a high degree of variation in the estimated values of the parameters between individuals for each hypothesis. A part of this variation may stem from physiological differences. For example, in the case of patient F, for whom particularly extreme values for some parameters were found, this can be linked to this patient being the only one with simultaneous activation of anti-VP and anti-sLT immune responses: Analyses suggested that the latter response could have a saturating effect, rendering the former irrelevant for the viral dynamics.

A relevant aspect of the model is that the dynamics of the immune response and their dependence on viral load were not explicitly modelled. The influence of the immune response on viral load is taken into account but the hypothetical contribution of BKV viral load to the building of an immune reaction is not addressed. This approach was chosen due to the high complexity and heterogeneity of the dynamics of immune reaction after immunosuppressant switch—especially the VP-sLT delay. Given that the mechanisms underlying this delay are currently unknown, we consider it to be highly unlikely that using currently available knowledge the immune response can be predicted from viral load.

The findings of our work on immune modes of action are especially relevant for future immunotherapeutic approaches against BKVN, since they suggest that the immune response against regulatory sLT antigens is central for BKV clearance. The use of T cells specific for BKV regulatory antigens is an interesting clinical approach, which has recently been shown to be technically possible [35]. In this study, the authors established a protocol for the ex-vivo generation of T cells specific for the antigens VP1 and LT, offering evidence of the specificity and safety of these cells [35].

Our BKV clearance modelling approach provides a framework for the hypothesis generation on the interrelations between cellular immunity and viral load at a personalised basis. Further research with the model could help us to improve therapeutic approaches in patients with BKVN, with the final aim of preventing kidney graft failure. The results of our model strongly suggest a general association between different target antigens and distinct mechanisms of the cellular immune system, linking structural VP antigens with the blockage of viral production and regulatory sLT antigens with cytotoxic effects. It further highlights the essential role of anti-sLT antigen response in clearance. These results should serve as a stimulus for further research on the differences between anti-VP and anti-sLT responses, particularly on their mechanisms, exploring possible physiological differences between patients in this respect. A suggested method could involve complementing the Elispot analysis with flow cytometry analysis of different cell populations reacting to each antigen (e.g. CD4^+^, CD8^+^, T helper 17, T regulatory) at all time points of the clearance process, with a special emphasis on the differences between the early- and late-stage responses. The knowledge gained through these experiments, as well as further implementations of our model, could open the door to the use of immunotherapy in the treatment, and perhaps prevention, of BKVN. Modelling approaches built upon our work could then be used in a personalised basis to tailor the therapy according to the characteristics of their viral and immune dynamics.

## Materials and methods

### Ethics statement

This study was approved by our local ethical review committee in compliance with the declaration of Helsinki. Informed consent was obtained from all patients (Ethic Committee Charité University Medicine, Berlin, Germany, 126/2001, 07/30/2001).

### Monitoring of BKVN patients

Patients were monitored for serum BKV viral load from 4/2006 to 9/2012 and for BKV specific immune response against VP and sLT from 01/2008 to 07/2010 as described in our previous study [16]. Screening for viral load was performed monthly over the first six months after kidney transplantation, then every three months, and again monthly during active BKV reactivation, while screening for specific immune response with Elispot was performed monthly since approximately the change of immunosuppressive therapy, until BKV clearance (<3000 copies·mL^-1^). A total of 167 viral load samples and 98 Elispot samples were collected. BKVN was confirmed by histological examination of the graft biopsy.

### Screening of BKV viral load

BKV viral load was measured by qPCR as described previously [15]. Briefly, BKV viral load was measured with TaqMan Real Time PCR. DNA was isolated from serum using a QIAamp DNA Mini Kit (Qiagen Corp., Hilden, Germany) according to the instructions of the manufacturer. PCR was performed with the TaqMan platform (ABI). PCR amplifications were set up in a reaction volume of 25 u/μL using primer and probe at final concentrations of 900 nM and 5 μM, respectively, amplifying the VP1 region of BKV. A plasmid standard containing the VP1 coding region of respective virus was used to determine the copy number per millilitre. Thermal cycling was begun with an initial denaturation step at 95°C for 10 min that was followed by 40 cycles at 95°C for 15 s and 60°C for 1 min.

### Screening of anti-BKV immune reaction

BKV-specific T cell immune response was determined by IFN-γ Elispot upon stimulation of PBMC with 5 different BKV proteins (VP1, VP2, VP3, st and LT) as described in our previous study [16]. Briefly, PBMC were isolated from 10–20 mL of heparinised blood using the standard Ficoll Hypaque density gradient centrifugation technique. For the Elispot assay, 96-well multiscreen filter plates (MAIPS 4510, Millipore, Billerica, MA, USA) were coated with 100 μL of primary IFN-γ monoclonal antibody (mAb) at a concentration of 3 μg/mL (IFNG M700A, Endogen, Woburn, MA, USA) and incubated overnight at 4°C. A standardised responder T-cell number of 2.5 × 10^5^ PBMC per well was added in quadruple or at least triplicate wells with one of the five stimulating peptides (1 μg/mL). Staphylococcus enterotoxin B (SEB; Sigma, Munich, Germany, 1 μg/mL) was used as positive control and negative controls were run in parallel using responder cells plus medium alone. Probes were incubated for 24 hours at 37°C. The detection of IFN-γ took place after an overnight incubation at 4°C with 100 μL (1 μL/mL) biotinylated detection IFN-γ antibody (IFNG-M701-B Biotin, Endogen). After adding streptavidine (1 μg/mL) for 2 hours at room temperature, spots were developed by adding 200μL visualization solution, AEC (3-amino-9-ethylcarbazole, Sigma) in acetate buffer supplemented with H_2_O_2_ 30% for 3–5 min. Resulting spots were counted using a computer-assisted Elispot reader (Immunospot, Cellular Technologies, Ltd., Cleveland, OH, USA). The number of SFU·10^−6^ PBMC was calculated by adding spot counts from each well.

### Parameter estimation of the mathematical models

The models were fitted using the function fminsearch of the mathematical open-access software Scilab, which employs the Nelder-Mead algorithm [36]. To ensure that the minimum of the objective function is reached, several replications (> 100) of the estimation were performed, using vastly different (> 2 orders of magnitude in some cases) starting parameter sets. The objective functions for the immune dynamics and the viral dynamics, which take the form of vertical least-squares, are defined in the Results section (Eqs 2 and 7).

To avoid overestimating the degrees of freedom of each hypothesis, parameters appearing only as the product of two free parameters are considered as only one free parameter. This is the case for model VPμ-sLTμ, where *m·max*_*μ*_ and *m·max*_*M*_ are estimated as two parameters, instead of three parameters.

### Model selection

Bayesian Information Criterion (BIC) differences were employed as the model selection criterion. Additionally, the Akaike’s Information Criterion (AIC) was also calculated. The corrected Akaike’s Information Criterion (AICc) was not used, as its value was not calculable for certain patient/hypotheses combinations. BIC and AIC were estimated for each patient *i* and hypothesis *h* under the assumption of independent, normally distributed errors

$${BIC}_{ih}\;=\;N_{i}\text{ln}\;f_{ih}+K_{ih}\text{ln}\;N_{i}$$

$${AIC}_{ih}\;=\;N_{i}\text{ln}\;f_{ih}+2K_{ih}$$ (8)

where *N*_*i*_ is the total number of measurements per patient *i*, *K*_*ih*_ is the number of parameters for patient *i* and hypothesis *h*, and *f*_*ih*_ is the objective function for patient *i* and hypothesis *h* as defined in Eq 6. [33] AIC and BIC differences were calculated as

$$\Delta {BIC}_{ih}\;=\;{BIC}_{ih}-min({BIC}_{i\;})$$

$$\Delta {AIC}_{ih}\;=\;{AIC}_{ih}-min({AIC}_{i\;})$$ (9)

where the function min denotes the lowest AIC or BIC achieved for a patient. A difference in the range [0, 2] for *ΔBIC*_*ih*_ is considered to give substantial empirical support for the hypothesis *h* in patient *i* [33].

### Estimation of 95% confidence intervals

95% confidence intervals were estimated using bootstrapping, as described in Banks *et al*. [37]. Briefly, for each of the six patients the dynamics were simulated with the best-performing hypothesis (VPε-sLTμ) and the best-fitting parameter set (Table 4). Residuals for the viral load were calculated as the difference between predicted and observed viral load for each time point. The residuals of each patient (excluding the first residual, which is zero by definition) were randomly resampled with replacement 1000 times, constructing 1000 artificial data sets for each patient, each with the same number of measurements as the patient. These artificial data sets were subject to fitting using as initial parameter values those in Table 4. The obtained distribution of estimated parameters for each patient was employed to calculate the 95% confidence intervals: for a normal distribution of parameter values for a patient, the confidence intervals were calculated as the mean ± 1.96 · standard deviation; for skewed distributions (absolute value of skewness or kurtosis higher than 2), the 95% confidence intervals were calculated directly from the 25^th^ and 975^-th^ entries in the set of ordered parameter estimates.

## Supporting information

S1 Table Parameters for the immune function curve. Results of the fitting for the immune response model in Eq 1 for all six patients and five antigens.
(PDF)

S2 Table Hypotheses on the dominant modes of action of the immune system as defined by the model. Description of the possible hypotheses on the dominant modes of action of the immune response against VP and sLT antigens, as defined by the model (Eqs 3–5).
(PDF)

S3 Table Detailed results for the model comparison criteria of the fittings for the nine hypotheses. The results for patient F and hypothesis VPε-sLTμ are shown additionally under the special assumption of a saturating sLT response.
(PDF)

S4 Table Results of the sensitivity analysis for the fixed parameters. (PDF)

S1 Fig Comparison of anti-VP responses fittings for patient A. Results of the fitting assuming only one activation event, compared to the fitting for two activation events.
(TIF)

S2 Fig Comparison of the fittings of the hypotheses to the best-performing hypothesis. The hypotheses are shown in order of increasing *f*_SUM_
(PDF)

S3 Fig Plotting of the sensitivity analysis results for the extreme parameter values. Note that for *k* only *k* = 10.7 was plotted, as *k* = 0.107 is not biologically meaningful.
(PDF)
