# Supplementary material for: Differential T cell response against BK virus regulatory and structural antigens: A viral dynamics modelling approach
Source: PLoS Comput Biol. 2018 May 10;14(5):e1005998. doi: 10.1371/journal.pcbi.1005998 (PMC5944912; doi:10.1371/journal.pcbi.1005998)
Supplement: S1 Table — Results of the fitting for the immune response model in Eq 1 for all six patients and five antigens. (PDF) [file pcbi.1005998.s001.pdf]

Antigens	Parameters	Patients					
		A[a]	B	C	D	E	F
VP1	t_{VP1}	0.00<br>$3.49\cdot10^{2}$	13.4	0.00	$7.05\cdot10^{-1}$	0.00	0.00
	r_{VP1}	$8.06\cdot10^{-2}$<br>$1.48\ \cdot10^{-1}$	$3.19\cdot10^{-2}$	$2.74\cdot10^{-2}$	$1.18\cdot10^{-1}$	$7.94\cdot10^{-2}$	$3.03\cdot10^{-1}$
	$max_{antiVP1}$	79.0<br>$2.58\cdot10^{2}$	$1.86\cdot10^{2}$	$1.47\cdot10^{2}$	$1.50\cdot10^{2}$	$2.70\cdot10^{2}$	$5.02\cdot10^{2}$
	dec_{VP1}	0.00<br>$2.62\cdot10^{-4}$	0.00	0.00	0.00	0.00	$5.76\cdot10^{-3}$
VP2	t_{VP2}	0.00<br>$3.14\cdot10^{2}$	0.00	0.00	$1.17\cdot10^{-1}$	22.7	0.00
	r_{VP2}	$9.05\cdot10^{-2}$<br>$1.18\ \cdot10^{-1}$	$5.73\cdot10^{-2}$	$2.28\cdot10^{-2}$	$1.01\cdot10^{-1}$	$5.89\cdot10^{-1}$	$2.89\cdot10^{-1}$
	$max_{antiVP2}$	51.6<br>$1.73\cdot10^{2}$	$1.28\cdot10^{2}$	$1.05\cdot10^{2}$	71.4	$1.65\cdot10^{2}$	$4.44\cdot10^{2}$
	dec_{VP2}	0.00<br>$2.04\cdot10^{-4}$	$1.15\cdot10^{-5}$	0.00	$8.69\cdot10^{-4}$	0.00	$5.32\cdot10^{-3}$
VP3	t_{VP3}	0.00<br>$3.17\cdot10^{2}$	$2.71\cdot10^{-2}$	0.00	$4.38\cdot10^{-1}$	26.7	0.00
	r_{VP3}	$8.48\cdot10^{-2}$<br>$1.21\ \cdot10^{-1}$	$5.69\cdot10^{-2}$	$1.31\cdot10^{-2}$	$1.55\cdot10^{-2}$	$1.27\cdot10^{2}$	$3.16\cdot10^{-1}$
	$max_{antiVP3}$	68.8	$1.43\cdot10^{2}$	$1.25\cdot10^{2}$	$6.30\cdot10^{4}$	$1.65\cdot10^{2}$	$5.02\cdot10^{2}$

		$2.88\cdot10^{2}$					
	dec_{VP3}	0.00	$7.52\cdot10^{-10}$	0.00	$1.70\cdot10^{-6}$	$2.41\cdot10^{-3}$	$5.99\cdot10^{-3}$
		$7.56\cdot10^{-4}$					
st	t_{st}	$3.19\cdot10^{2}$	$1.85\cdot10^{2}$	-	57.0	28.0	0.00
	r_{st}	$1.34\cdot10^{-1}$	$8.36\cdot10^{-1}$	-	1.25	$1.06\cdot10^{-1}$	$6.16\cdot10^{-1}$
	max_{antist}	$2.01\cdot10^{2}$	$1.66\cdot10^{2}$	-	$1.29\cdot10^{2}$	$2.95\cdot10^{2}$	$4.19\cdot10^{2}$
	dec_{st}	$7.60\cdot10^{-6}$	$1.22\cdot10^{-4}$	-	$1.84\cdot10^{-5}$	0.00	$5.80\cdot10^{-3}$
LT	t_{LT}	$3.27\cdot10^{2}$	$1.81\cdot10^{2}$	-	44.6	1.13	0.00
	r_{LT}	$1.66\cdot10^{-1}$	$1.18\cdot10^{-1}$	-	1.17	$9.63\cdot10^{-2}$	1.66
	max_{antiLT}	$1.70\cdot10^{2}$	$2.02\cdot10^{2}$	-	$1.55\cdot10^{2}$	$4.30\cdot10^{2}$	$1.69\cdot10^{2}$
	dec_{LT}	$1.00\cdot10^{-6}$	$1.08\cdot10^{-5}$	-	$3.82\cdot10^{-3}$	$6.53\cdot10^{-3}$	$3.46\cdot10^{-3}$
f	**Obj. Function**	$\mathbf{4.16\cdot10^{-2}}$	$\mathbf{3.99\cdot10^{-1}}$	$\mathbf{8.21\cdot10^{-1}}$	$\mathbf{3.84\cdot10^{-2}}$	$\mathbf{8.30\cdot10^{-3}}$	$\mathbf{3.70\cdot10^{-3}}$

Results of the fitting for the immune response model in Eq. 1 for all six patients and five antigens. The last row indicates the achieved value of the objective function (Eq. 2).

[a]For Patient A, the response against the VP antigens was described using a second activation time t_{a2} with a second parameter set to achieve a better fit.
