# Supplementary material for: Differential T cell response against BK virus regulatory and structural antigens: A viral dynamics modelling approach
Source: PLoS Comput Biol. 2018 May 10;14(5):e1005998. doi: 10.1371/journal.pcbi.1005998 (PMC5944912; doi:10.1371/journal.pcbi.1005998)
Supplement: S2 Table — Description of the possible hypotheses on the dominant modes of action of the immune response against VP and sLT antigens, as defined by the model (Eqs 3–5). (PDF) [file pcbi.1005998.s002.pdf]

Hypothesis	Definition	Description
VPε-sLTε	$max_\mu = max_M = max_\upsilon = max_N = 0$<br>$0 < max_\varepsilon < 1$<br>$0 < max_E < 1$	Anti-VP response and anti-sLT response both trigger blockage of virus production
VPε-sLTμ	$max_E = max_\mu = max_\upsilon = max_N = 0$<br>$max_\varepsilon = 1$<br>$max_M = 1$	Anti-VP response triggers blockage of virus production<br>Anti-sLT response triggers accelerated killing of infected cells
VPε-sLTυ	$max_E = max_\mu = max_M = max_\upsilon = 0$<br>$max_\varepsilon = 1$<br>$max_N = 1$	Anti-VP response triggers blockage of virus production<br>Anti-sLT response triggers blockage of cell infection
VPμ-sLTε	$max_\varepsilon = max_M = max_\upsilon = max_N = 0$<br>$max_\mu = 1$<br>$max_E = 1$	Anti-VP response triggers accelerated killing of infected cells<br>Anti-sLT response triggers blockage of virus production
VPμ-sLTμ	$max_\varepsilon = max_E = max_\upsilon = max_N = 0$<br>$0 < max_\mu < 1$<br>$0 < max_M < 1$	Anti-VP response and anti-sLT response both trigger accelerated killing of infected cells
VPμ-sLTυ	$max_\varepsilon = max_E = max_M = max_\upsilon = 0$<br>$max_\mu = 1$<br>$max_N = 1$	Anti-VP response triggers accelerated killing of infected cells<br>Anti-sLT response triggers blockage of cell infection
VPυ-sLTε	$max_\varepsilon = max_\mu = max_M = max_N = 0$<br>$max_\upsilon = 1$<br>$max_E = 1$	Anti-VP response triggers blockage of cell infection<br>Anti-sLT response triggers blockage of virus production
VPυ-sLTμ	$max_\varepsilon = max_E = max_\mu = max_N = 0$<br>$max_\upsilon = 1$<br>$max_M = 1$	Anti-VP response triggers blockage of cell infection<br>Anti-sLT response triggers accelerated killing of infected cells
VPυ-sLTυ	$max_\varepsilon = max_E = max_M = max_\mu = 0$<br>$0 < max_\upsilon < 1$	Anti-VP and anti-sLT response both trigger blockage of cell infection

	$0 < max_N < 1$	

Description of the possible hypotheses on the dominant mechanisms of the immune response against VP and sLT antigens, as defined by the model (Eqs. 3-5).
