# Supplementary material for: Differential T cell response against BK virus regulatory and structural antigens: A viral dynamics modelling approach
Source: PLoS Comput Biol. 2018 May 10;14(5):e1005998. doi: 10.1371/journal.pcbi.1005998 (PMC5944912; doi:10.1371/journal.pcbi.1005998)
Supplement: S3 Table — The results for patient F and hypothesis VPε-sLTμ are shown additionally under the special assumption of a saturating sLT response. (PDF) [file pcbi.1005998.s003.pdf]

| Patient | Measurement | VPε-sLTε | VPε-sLTμ | VPε-sLTν | VPμ-sLTε | VPμ-sLTμ | VPμ-sLTν | VPν-sLTε | VPν-sLTμ | VPν-sLTν | Number Measurements | VPε-sLTμ-Saturated. |
|---|---|---|---|---|---|---|---|---|---|---|---|---|
| **No. of parameters** | | 7 | 6 | 5 | 6 | 7 | 6 | 5 | 6 | 7 | | 4 |
| **No. of parameters Patient C** | | 4 | 3 | 3 | 4 | 4 | 4 | 3 | 3 | 4 | | |
| A | RSS | 0.83700 | **0.21630** | 0.46290 | 0.38430 | 0.30860 | 0.42640 | 1.02090 | 0.35370 | 13.17120 | 7 | |
| | *f* | 0.11957 | 0.03090 | 0.06613 | 0.05490 | 0.04409 | 0.06091 | 0.14584 | 0.05053 | 1.88160 | | |
| | BIC | -1.24552 | -12.66353 | -9.28353 | -8.64023 | -8.22997 | -7.91255 | -3.74703 | -9.22105 | 18.04623 | | |
| | ΔBIC | 11.41801 | **0.00000** | 3.38000 | 4.02330 | 4.43357 | 4.75098 | 8.91650 | 3.44248 | 30.70976 | | |
| | AIC | -0.86689 | -12.33899 | -9.01308 | -8.31569 | -7.85134 | -7.58801 | -3.47658 | -8.89651 | 18.42486 | | |
| | ΔAIC | 11.47210 | **0.00000** | 3.32591 | 4.02330 | 4.48766 | 4.75098 | 8.86241 | 3.44248 | 30.76385 | | |
| B | RSS | 0.47900 | **0.07840** | 0.42320 | 0.22070 | 0.15330 | 0.15630 | 0.43110 | 0.13960 | 0.43590 | 7 | |
| | *f* | 0.06843 | 0.01120 | 0.06046 | 0.03153 | 0.02190 | 0.02233 | 0.06159 | 0.01994 | 0.06227 | | |
| | BIC | -5.15238 | -19.76743 | -9.91119 | -12.52257 | -13.12751 | -14.93776 | -9.78173 | -15.72873 | -5.81240 | | |
| | ΔBIC | 14.61505 | **0.00000** | 9.85624 | 7.24486 | 6.63992 | 4.82967 | 9.98570 | 4.03870 | 13.95503 | | |
| | AIC | -4.77375 | -19.44289 | -9.64074 | -12.19803 | -12.74888 | -14.61322 | -9.51128 | -15.40419 | -5.43377 | | |
| | ΔAIC | 14.66914 | **0.00000** | 9.80215 | 7.24486 | 6.69401 | 4.82967 | 9.93161 | 4.03870 | 14.00912 | | |
| C | RSS | 0.17920 | 0.14420 | 0.17220 | 0.14980 | 0.14980 | 0.14980 | 0.14700 | 0.14700 | **0.14280** | 14 | |
| | *f* | 0.01280 | 0.01030 | 0.01230 | 0.01070 | 0.01070 | 0.01070 | 0.01050 | 0.01050 | 0.01020 | | |
| | BIC | -50.46011 | -56.14139 | -53.65701 | -55.60799 | -52.96893 | -55.60799 | -55.87215 | -55.87215 | -53.63892 | | |
| | ΔBIC | **5.68128** | **0.00000** | 2.48438 | **0.53340** | 3.17246 | **0.53340** | **0.26924** | **0.26924** | 2.50247 | | |
| | AIC | -53.01634 | -58.05856 | -55.57418 | -57.52516 | -55.52516 | -57.52516 | -57.78932 | -57.78932 | -56.19515 | | |
| | ΔAIC | **5.04222** | **0.00000** | 2.48438 | **0.53340** | **2.53340** | **0.53340** | **0.26924** | **0.26924** | 1.86341 | | |
| D | RSS | **0.00020** | 0.00174 | 0.43690 | 0.04320 | 0.74250 | 0.63700 | 0.07480 | 0.52190 | 9.35600 | 4 | |
| | *f* | 0.00005 | 0.00044 | 0.10923 | 0.01080 | 0.18563 | 0.15925 | 0.01870 | 0.13048 | 2.33900 | | |
| | BIC | -29.85862 | -22.64289 | -1.92591 | -9.79507 | 2.96795 | 0.96865 | -8.98546 | 0.17147 | 13.10295 | | |
| | ΔBIC | **0.00000** | 7.21573 | 27.93271 | 20.06355 | 32.82657 | 30.82727 | 20.87316 | 30.03009 | 42.96157 | | |
| | AIC | -25.56268 | -18.96066 | 1.14262 | -6.11284 | 7.26389 | 4.65088 | -5.91693 | 3.85371 | 17.39889 | | |
| | ΔAIC | **0.00000** | 6.60202 | 26.70530 | 19.44984 | 32.82657 | 30.21356 | 19.64575 | 29.41639 | 42.96157 | | |
| E | RSS | 1.38510 | 0.44730 | 2.05310 | **0.40660** | 0.69740 | 0.88140 | 24.28010 | 2.30030 | 19.20330 | 8 | |
| | *f* | 0.17314 | 0.05591 | 0.25664 | 0.05083 | 0.08718 | 0.11018 | 3.03501 | 0.28754 | 2.40041 | | |
| | BIC | 0.52674 | -10.59509 | -0.48352 | -11.35829 | -4.96261 | -5.16883 | 19.27893 | 2.50543 | 21.56122 | | |
| | ΔBIC | 11.88502 | **0.76320** | 10.87477 | **0.00000** | 6.39568 | 6.18945 | 30.63722 | 13.86372 | 32.91950 | | |

| | | | | | | | | | | | | |
|---|---|---|---|---|---|---|---|---|---|---|---|---|
| | AIC | -0.02935 | -11.07174 | -0.88073 | -11.83494 | -5.51870 | -5.64548 | 18.88172 | 2.02878 | 21.00512 | | |
| | ΔAIC | 11.80558 | **0.76320** | 10.95421 | **0.00000** | 6.31623 | 6.18945 | 30.71666 | 13.86372 | 32.84006 | | |
| F | RSS | 5.02810 | 0.62390 | 5.24450 | 0.99700 | 0.85260 | **0.48240** | 5.25820 | 0.48250 | 17.01750 | | 0.65240 |
| | $f$ | 1.25703 | 0.15598 | 1.31113 | 0.24925 | 0.21315 | 0.12060 | 1.31455 | 0.12063 | 4.25438 | | 0.16310 |
| | BIC | 10.61905 | 0.88553 | 8.01501 | 2.76057 | 3.52102 | -0.14334 | 8.02545 | -0.14251 | 15.49585 | | -1.70839 |
| | ΔBIC | 10.76239 | **1.02887** | 8.15835 | 2.90391 | 3.66436 | **0.00000** | 8.16879 | **0.00083** | 15.63919 | 4 | **-1.56505** |
| | AIC | 14.91499 | 4.56776 | 11.08354 | 6.44280 | 7.81696 | 3.53890 | 11.09398 | 3.53973 | 19.79179 | | 0.4643 |
| | ΔAIC | 11.37610 | **1.02887** | 7.54465 | 2.90391 | 4.27807 | **0.00000** | 7.55508 | **0.00083** | 16.25290 | | **-2.79246** |
| $f_{SUM}$ | | 1.63101 | **0.26472** | 1.81587 | 0.40800 | 0.56264 | 0.48397 | 4.58619 | 0.61961 | 10.94786 | | |
| Median ΔBIC | | 11.09020 | **0.38160** | 9.00729 | 3.46360 | 5.41462 | 4.79033 | 9.45110 | 3.74059 | 23.17448 | | |
| Median ΔAIC | | 11.42410 | **0.38160** | 8.67340 | 3.46360 | 5.40195 | 4.79033 | 9.39701 | 3.74059 | 23.50837 | | |

The results for the residual sum of squares (RSS), the objective function $f$ (Eq. 6), BIC, ΔBIC, AIC and ΔAIC (Eq. 8 and 9) are shown for each one of the hypotheses and patients; as well as for a special case of the VPε-sLTμ for patient F, assuming a saturating anti-sLT response. The sum of the objective functions over all patients is shown as $f_{SUM}$. In bold are highlighted: The lowest per patient values for $f$, as well as the scores of ΔBIC and ΔAIC within the range of substantial empirical support (<2). The definitions of the hypotheses are shown in S2 Table.
