# Supplementary material for: Differential T cell response against BK virus regulatory and structural antigens: A viral dynamics modelling approach
Source: PLoS Comput Biol. 2018 May 10;14(5):e1005998. doi: 10.1371/journal.pcbi.1005998 (PMC5944912; doi:10.1371/journal.pcbi.1005998)
Supplement: S4 Table — (PDF) [file pcbi.1005998.s004.pdf]

Results Sensitivity Analysis

The results for the sensitivity analysis of the fixed parameters (*d*, *p*, *g*, *ß*, and *k*) are reproduced below. The results for the original values of the parameters are marked in red. The sensitivity analysis was on a one-factor-at-a-time principle, with the values shown on the first row of each table. *f* is the lowest value of the objective function (Eq. 6) achieved for each patient and value of the parameter, f_{SUM} indicates the sum of the objective functions for all six patients.

**Value *g***	**$1.00\cdot10^{-1}$**	**$1.58\cdot10^{-1}$**	**$2.51\cdot10^{-1}$**	**$3.98\cdot10^{-1}$**	**$6.31\cdot10^{-1}$**	**1.00**	**1.58**	**2.51**	**3.98**	**6.31**	**10.0**
f_A	$3.12\cdot10^{-2}$	$3.40\cdot10^{-2}$	$3.40\cdot10^{-2}$	$2.99\cdot10^{-2}$	$3.40\cdot10^{-2}$	$3.09\cdot10^{-2}$	$3.00\cdot10^{-2}$	$3.12\cdot10^{-2}$	$3.22\cdot10^{-2}$	$3.40\cdot10^{-2}$	$3.19\cdot10^{-2}$
f_B	$1.64\cdot10^{-2}$	$1.56\cdot10^{-2}$	$1.64\cdot10^{-2}$	$1.64\cdot10^{-2}$	$1.64\cdot10^{-2}$	$1.12\cdot10^{-2}$	$1.12\cdot10^{-2}$	$1.64\cdot10^{-2}$	$1.64\cdot10^{-2}$	$1.64\cdot10^{-2}$	$1.58\cdot10^{-2}$
f_C	$1.03\cdot10^{-2}$	$1.27\cdot10^{-2}$	$1.27\cdot10^{-2}$	$1.27\cdot10^{-2}$	$1.27\cdot10^{-2}$	$1.03\cdot10^{-2}$	$1.27\cdot10^{-2}$	$1.27\cdot10^{-2}$	$1.27\cdot10^{-2}$	$1.27\cdot10^{-2}$	$1.27\cdot10^{-2}$
f_D	$5.40\cdot10^{-3}$	$5.40\cdot10^{-3}$	$5.40\cdot10^{-3}$	$5.40\cdot10^{-3}$	$5.40\cdot10^{-3}$	$4.30\cdot10^{-3}$	$4.30\cdot10^{-3}$	$5.40\cdot10^{-3}$	$5.40\cdot10^{-3}$	$5.40\cdot10^{-3}$	$5.40\cdot10^{-3}$
f_E	$5.77\cdot10^{-2}$	$5.77\cdot10^{-2}$	$5.77\cdot10^{-2}$	$5.77\cdot10^{-2}$	$5.77\cdot10^{-2}$	$5.59\cdot10^{-2}$	$5.59\cdot10^{-2}$	$5.77\cdot10^{-2}$	$5.77\cdot10^{-2}$	$5.77\cdot10^{-2}$	$5.77\cdot10^{-2}$
f_F	$2.04\cdot10^{-1}$	$1.75\cdot10^{-1}$	$2.12\cdot10^{-1}$	$2.12\cdot10^{-1}$	$1.84\cdot10^{-1}$	$1.56\cdot10^{-1}$	$1.56\cdot10^{-1}$	$2.12\cdot10^{-1}$	$1.75\cdot10^{-1}$	$2.12\cdot10^{-1}$	$1.56\cdot10^{-1}$
f_{SUM}	**$3.25\cdot10^{-1}$**	**$3.01\cdot10^{-1}$**	**$3.38\cdot10^{-1}$**	**$3.34\cdot10^{-1}$**	**$3.11\cdot10^{-1}$**	**$2.69\cdot10^{-1}$**	**$2.70\cdot10^{-1}$**	**$3.36\cdot10^{-1}$**	**$2.99\cdot10^{-1}$**	**$3.38\cdot10^{-1}$**	**$2.80\cdot10^{-1}$**

**Value *d***	**$1.00\cdot10^{-3}$**	**$1.58\cdot10^{-3}$**	**$2.51\cdot10^{-3}$**	**$3.98\cdot10^{-3}$**	**$6.31\cdot10^{-3}$**	**$1.00\cdot10^{-2}$**	**$1.58\cdot10^{-2}$**	**$2.51\cdot10^{-2}$**	**$3.98\cdot10^{-2}$**	**$6.31\cdot10^{-2}$**	**$1.00\cdot10^{-1}$**
f_A	$4.21\cdot10^{-2}$	$6.16\cdot10^{-2}$	$4.16\cdot10^{-2}$	$5.76\cdot10^{-2}$	$4.30\cdot10^{-2}$	$3.09\cdot10^{-2}$	$2.93\cdot10^{-2}$	$4.29\cdot10^{-2}$	$4.19\cdot10^{-2}$	$3.55\cdot10^{-2}$	$4.17\cdot10^{-2}$
f_B	$1.60\cdot10^{-2}$	$1.31\cdot10^{-2}$	$1.22\cdot10^{-2}$	$1.58\cdot10^{-2}$	$1.47\cdot10^{-2}$	$1.12\cdot10^{-2}$	$1.16\cdot10^{-2}$	$2.92\cdot10^{-2}$	$2.64\cdot10^{-2}$	$3.85\cdot10^{-2}$	$3.84\cdot10^{-2}$
f_C	$2.55\cdot10^{-2}$	$2.12\cdot10^{-2}$	$1.57\cdot10^{-2}$	$3.46\cdot10^{-2}$	$1.27\cdot10^{-2}$	$1.03\cdot10^{-2}$	$1.11\cdot10^{-2}$	$1.27\cdot10^{-2}$	$1.28\cdot10^{-2}$	$1.29\cdot10^{-2}$	$1.30\cdot10^{-2}$
f_D	$5.50\cdot10^{-3}$	$5.70\cdot10^{-3}$	$5.90\cdot10^{-3}$	$6.20\cdot10^{-3}$	$5.50\cdot10^{-3}$	$4.30\cdot10^{-3}$	$4.70\cdot10^{-3}$	$5.60\cdot10^{-3}$	$9.00\cdot10^{-3}$	$7.40\cdot10^{-3}$	$1.54\cdot10^{-2}$
f_E	$5.52\cdot10^{-2}$	$5.58\cdot10^{-2}$	$5.61\cdot10^{-2}$	$5.69\cdot10^{-2}$	$5.67\cdot10^{-2}$	$5.59\cdot10^{-2}$	$5.86\cdot10^{-2}$	$6.02\cdot10^{-2}$	$6.07\cdot10^{-2}$	$6.48\cdot10^{-2}$	$5.42\cdot10^{-2}$
f_F	$2.03\cdot10^{-1}$	$1.98\cdot10^{-1}$	$1.65\cdot10^{-1}$	$1.99\cdot10^{-1}$	$2.00\cdot10^{-1}$	$1.56\cdot10^{-1}$	$1.56\cdot10^{-1}$	$2.01\cdot10^{-1}$	$1.97\cdot10^{-1}$	$2.15\cdot10^{-1}$	$1.64\cdot10^{-1}$
f_{SUM}	**$3.47\cdot10^{-1}$**	**$3.55\cdot10^{-1}$**	**$2.97\cdot10^{-1}$**	**$3.70\cdot10^{-1}$**	**$3.32\cdot10^{-1}$**	**$2.69\cdot10^{-1}$**	**$2.72\cdot10^{-1}$**	**$3.52\cdot10^{-1}$**	**$3.47\cdot10^{-1}$**	**$3.74\cdot10^{-1}$**	**$3.27\cdot10^{-1}$**

**Value *p***	**1.50**	**2.38**	**3.77**	**5.97**	**9.46**	**15.0**	**23.8**	**37.7**	**59.7**	**94.6**	**$1.50\cdot10^{2}$**
f_A	$3.47\cdot10^{-2}$	$3.47\cdot10^{-2}$	$3.57\cdot10^{-2}$	$3.22\cdot10^{-2}$	$3.46\cdot10^{-2}$	$3.09\cdot10^{-2}$	$3.39\cdot10^{-2}$	$3.12\cdot10^{-2}$	$3.55\cdot10^{-2}$	$2.90\cdot10^{-2}$	$3.42\cdot10^{-2}$
f_B	$1.48\cdot10^{-2}$	$1.48\cdot10^{-2}$	$2.00\cdot10^{-2}$	$1.55\cdot10^{-2}$	$1.51\cdot10^{-2}$	$1.12\cdot10^{-2}$	$1.12\cdot10^{-2}$	$2.58\cdot10^{-2}$	$1.56\cdot10^{-2}$	$1.60\cdot10^{-2}$	$1.69\cdot10^{-2}$
f_C	$1.27\cdot10^{-2}$	$1.27\cdot10^{-2}$	$1.27\cdot10^{-2}$	$1.27\cdot10^{-2}$	$1.27\cdot10^{-2}$	$1.03\cdot10^{-2}$	$1.27\cdot10^{-2}$	$1.27\cdot10^{-2}$	$1.27\cdot10^{-2}$	$1.27\cdot10^{-2}$	$1.27\cdot10^{-2}$
f_D	$5.10\cdot10^{-3}$	$5.10\cdot10^{-3}$	$5.30\cdot10^{-3}$	$5.40\cdot10^{-3}$	$5.20\cdot10^{-3}$	$4.40\cdot10^{-3}$	$4.90\cdot10^{-3}$	$5.20\cdot10^{-3}$	$5.20\cdot10^{-3}$	$5.40\cdot10^{-3}$	$5.40\cdot10^{-3}$
f_E	$5.82\cdot10^{-2}$	$5.82\cdot10^{-2}$	$5.80\cdot10^{-2}$	$5.84\cdot10^{-2}$	$5.73\cdot10^{-2}$	$5.59\cdot10^{-2}$	$5.61\cdot10^{-2}$	$5.80\cdot10^{-2}$	$5.72\cdot10^{-2}$	$5.80\cdot10^{-2}$	$5.73\cdot10^{-2}$
f_F	$1.56\cdot10^{-1}$	$1.56\cdot10^{-1}$	$2.03\cdot10^{-1}$	$1.60\cdot10^{-1}$	$2.08\cdot10^{-1}$	$1.56\cdot10^{-1}$	$1.56\cdot10^{-1}$	$2.08\cdot10^{-1}$	$2.06\cdot10^{-1}$	$1.83\cdot10^{-1}$	$1.83\cdot10^{-1}$
f_{SUM}	**$3.47\cdot10^{-2}$**	**$3.47\cdot10^{-2}$**	**$3.57\cdot10^{-2}$**	**$3.22\cdot10^{-2}$**	**$3.46\cdot10^{-2}$**	**$3.09\cdot10^{-2}$**	**$3.39\cdot10^{-2}$**	**$3.12\cdot10^{-2}$**	**$3.55\cdot10^{-2}$**	**$2.90\cdot10^{-2}$**	**$3.42\cdot10^{-2}$**

**Value *ß***	**$3.00\cdot10^{-9}$**	**$4.75\cdot10^{-9}$**	**$7.54\cdot10^{-9}$**	**$1.19\cdot10^{-8}$**	**$1.89\cdot10^{-8}$**	**$3.00\cdot10^{-8}$**	**$4.75\cdot10^{-8}$**	**$7.54\cdot10^{-8}$**	**$1.19\cdot10^{-7}$**	**$1.89\cdot10^{-7}$**	**$3.00\cdot10^{-7}$**
f_A	$3.79\cdot10^{-2}$	$3.34\cdot10^{-2}$	$3.56\cdot10^{-2}$	$3.86\cdot10^{-2}$	$3.55\cdot10^{-2}$	$3.09\cdot10^{-2}$	$4.06\cdot10^{-2}$	$5.18\cdot10^{-2}$	$4.60\cdot10^{-2}$	$6.34\cdot10^{-2}$	$7.03\cdot10^{-2}$
f_B	$2.49\cdot10^{-2}$	$1.55\cdot10^{-2}$	$1.52\cdot10^{-2}$	$1.57\cdot10^{-2}$	$1.71\cdot10^{-2}$	$1.12\cdot10^{-2}$	$1.33\cdot10^{-2}$	$1.45\cdot10^{-2}$	$1.37\cdot10^{-2}$	$1.50\cdot10^{-2}$	$1.48\cdot10^{-2}$
f_C	$1.05\cdot10^{-2}$	$1.05\cdot10^{-2}$	$1.05\cdot10^{-2}$	$1.06\cdot10^{-2}$	$1.12\cdot10^{-2}$	$1.03\cdot10^{-2}$	$1.06\cdot10^{-2}$	$1.11\cdot10^{-2}$	$1.22\cdot10^{-2}$	$1.40\cdot10^{-2}$	$1.72\cdot10^{-2}$
f_D	$5.90\cdot10^{-3}$	$6.10\cdot10^{-3}$	$5.70\cdot10^{-3}$	$6.40\cdot10^{-3}$	$5.40\cdot10^{-3}$	$4.40\cdot10^{-3}$	$4.30\cdot10^{-3}$	$4.30\cdot10^{-3}$	$4.50\cdot10^{-3}$	$4.30\cdot10^{-3}$	$3.70\cdot10^{-3}$
f_E	$5.82\cdot10^{-2}$	$5.83\cdot10^{-2}$	$5.81\cdot10^{-2}$	$5.82\cdot10^{-2}$	$5.78\cdot10^{-2}$	$5.59\cdot10^{-2}$	$5.56\cdot10^{-2}$	$5.70\cdot10^{-2}$	$5.59\cdot10^{-2}$	$5.44\cdot10^{-2}$	$5.56\cdot10^{-2}$
f_F	$1.23\cdot10^{-1}$	$1.54\cdot10^{-1}$	$1.59\cdot10^{-1}$	$1.32\cdot10^{-1}$	$1.44\cdot10^{-1}$	$1.56\cdot10^{-1}$	$1.85\cdot10^{-1}$	$1.55\cdot10^{-1}$	$1.52\cdot10^{-1}$	$1.52\cdot10^{-1}$	$2.11\cdot10^{-1}$
f_{SUM}	**$2.60\cdot10^{-1}$**	**$2.78\cdot10^{-1}$**	**$2.85\cdot10^{-1}$**	**$2.62\cdot10^{-1}$**	**$2.71\cdot10^{-1}$**	**$2.69\cdot10^{-1}$**	**$3.09\cdot10^{-1}$**	**$2.94\cdot10^{-1}$**	**$2.85\cdot10^{-1}$**	**$3.03\cdot10^{-1}$**	**$3.73\cdot10^{-1}$**

**Value *k***	**$1.02\cdot10^{-1}$**	**$1.62\cdot10^{-1}$**	**$2.56\cdot10^{-1}$**	**$4.06\cdot10^{-1}$**	**$6.44\cdot10^{-1}$**	**1.02**	**1.62**	**2.56**	**4.06**	**6.44**	**10.2**
f_A	$7.25\cdot10^{-2}$	$4.20\cdot10^{-2}$	$5.11\cdot10^{-2}$	$5.86\cdot10^{-2}$	$4.28\cdot10^{-2}$	$3.09\cdot10^{-2}$	$2.93\cdot10^{-2}$	$4.03\cdot10^{-2}$	$3.42\cdot10^{-2}$	$4.62\cdot10^{-2}$	$2.91\cdot10^{-2}$
f_B	$1.73\cdot10^{-2}$	$1.65\cdot10^{-2}$	$1.65\cdot10^{-2}$	$1.69\cdot10^{-2}$	$1.44\cdot10^{-2}$	$1.12\cdot10^{-2}$	$1.16\cdot10^{-2}$	$1.36\cdot10^{-2}$	$3.87\cdot10^{-2}$	$1.73\cdot10^{-2}$	$3.06\cdot10^{-2}$
f_C	$2.49\cdot10^{-2}$	$2.07\cdot10^{-2}$	$1.58\cdot10^{-2}$	$3.39\cdot10^{-2}$	$1.27\cdot10^{-2}$	$1.03\cdot10^{-2}$	$1.12\cdot10^{-2}$	$1.27\cdot10^{-2}$	$1.08\cdot10^{-2}$	$1.07\cdot10^{-2}$	$1.07\cdot10^{-2}$
f_D	$5.30\cdot10^{-3}$	$5.30\cdot10^{-3}$	$2.80\cdot10^{-3}$	$5.60\cdot10^{-3}$	$5.60\cdot10^{-3}$	$4.30\cdot10^{-3}$	$4.80\cdot10^{-3}$	$6.00\cdot10^{-3}$	$5.30\cdot10^{-3}$	$1.51\cdot10^{-2}$	$3.00\cdot10^{-2}$
$f_{\cdot10}$	$5.53\cdot10^{-2}$	$5.58\cdot10^{-2}$	$5.58\cdot10^{-2}$	$5.63\cdot10^{-2}$	$5.68\cdot10^{-2}$	$5.59\cdot10^{-2}$	$5.89\cdot10^{-2}$	$6.01\cdot10^{-2}$	$6.21\cdot10^{-2}$	$5.99\cdot10^{-2}$	$6.28\cdot10^{-2}$
f_F	$1.56\cdot10^{-1}$	$2.02\cdot10^{-1}$	$2.05\cdot10^{-1}$	$2.01\cdot10^{-1}$	$1.56\cdot10^{-1}$	$1.56\cdot10^{-1}$	$1.56\cdot10^{-1}$	$1.99\cdot10^{-1}$	$1.64\cdot10^{-1}$	$1.72\cdot10^{-1}$	$1.58\cdot10^{-1}$
f_{SUM}	**$3.31\cdot10^{-1}$**	**$3.42\cdot10^{-1}$**	**$3.47\cdot10^{-1}$**	**$3.73\cdot10^{-1}$**	**$2.88\cdot10^{-1}$**	**$2.69\cdot10^{-1}$**	**$2.72\cdot10^{-1}$**	**$3.32\cdot10^{-1}$**	**$3.15\cdot10^{-1}$**	**$3.21\cdot10^{-1}$**	**$3.22\cdot10^{-1}$**
