# Supplementary figures and images for: Differential T cell response against BK virus regulatory and structural antigens: A viral dynamics modelling approach
Source: PLoS Comput Biol. 2018 May 10;14(5):e1005998. doi: 10.1371/journal.pcbi.1005998 (PMC5944912; doi:10.1371/journal.pcbi.1005998)

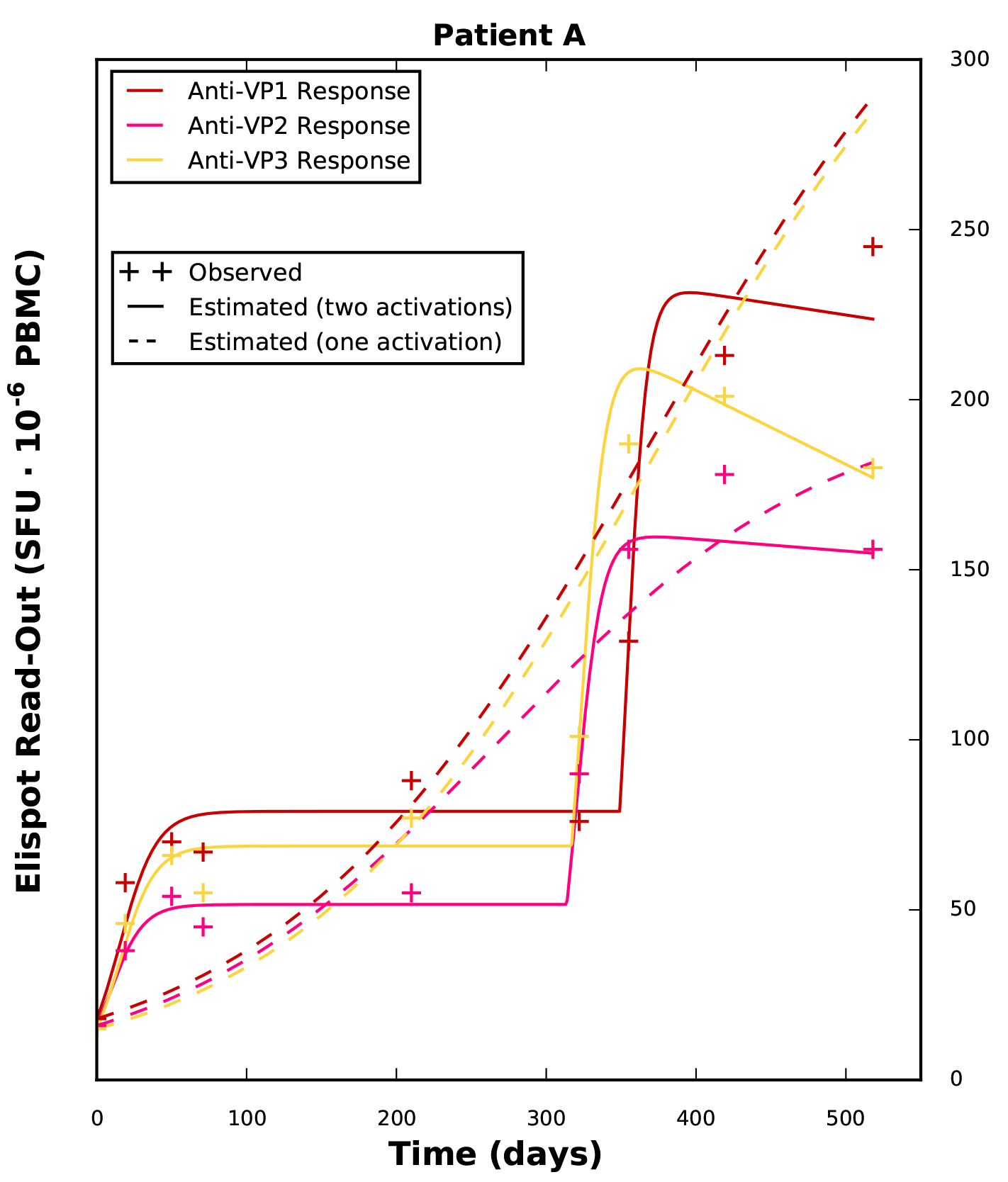

Supplement: S1 Fig — Results of the fitting assuming only one activation event, compared to the fitting for two activation events. (TIF) [file pcbi.1005998.s005.tif]
