# Supplementary material for: Differential T cell response against BK virus regulatory and structural antigens: A viral dynamics modelling approach
Source: PLoS Comput Biol. 2018 May 10;14(5):e1005998. doi: 10.1371/journal.pcbi.1005998 (PMC5944912; doi:10.1371/journal.pcbi.1005998)
Supplement: S2 Fig — The hypotheses are shown in order of increasing fSUM (PDF) [file pcbi.1005998.s006.pdf]

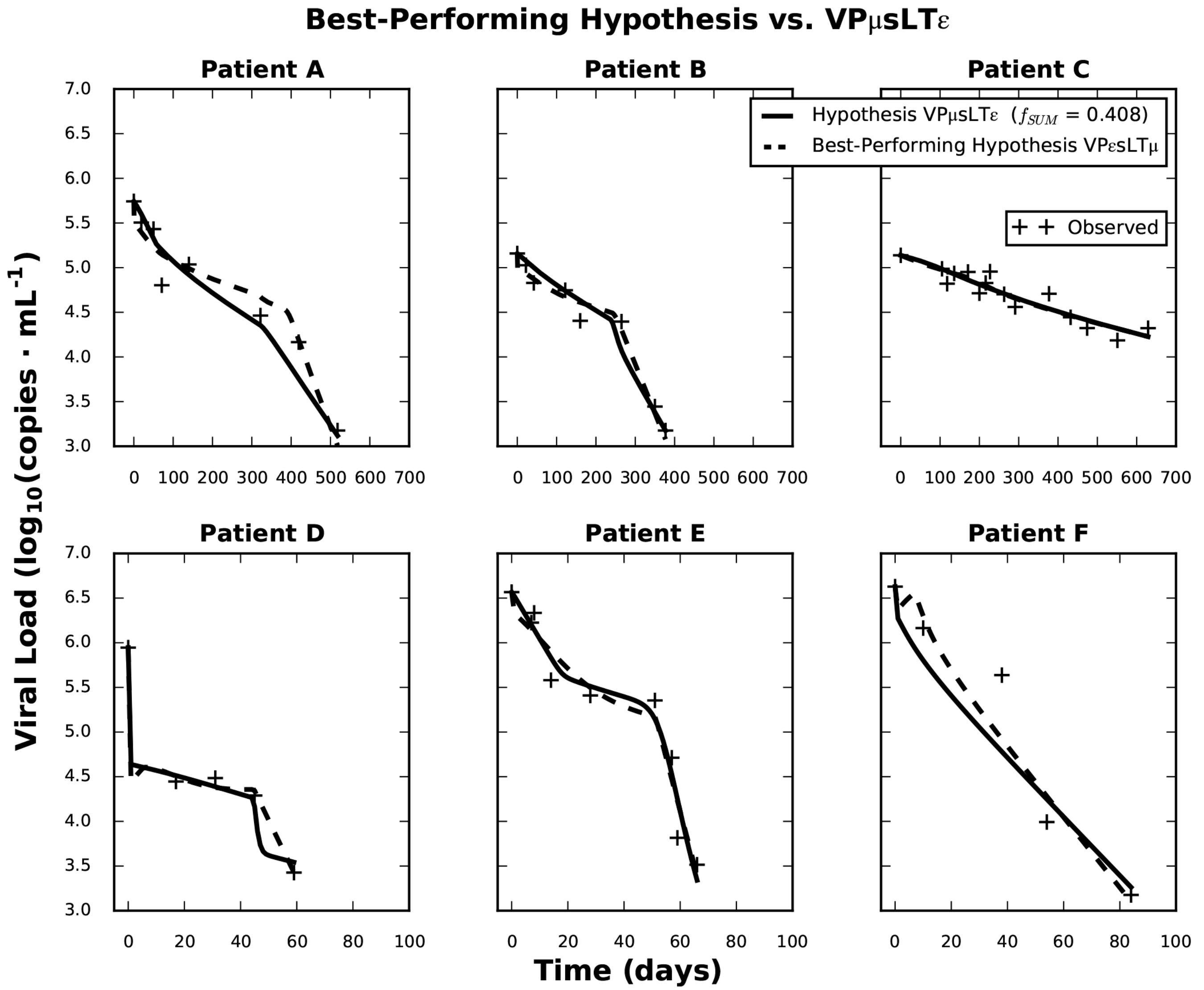

Best-Performing Hypothesis vs. VPμsLTε
Patient A
Patient B
Patient C
Patient D
Patient E
Patient F
Hypothesis VPμsLTε (f_{SUM} = 0.408)
Best-Performing Hypothesis VPεsLTμ
Observed
Viral Load (log10(copies · mL-1))
Time (days)

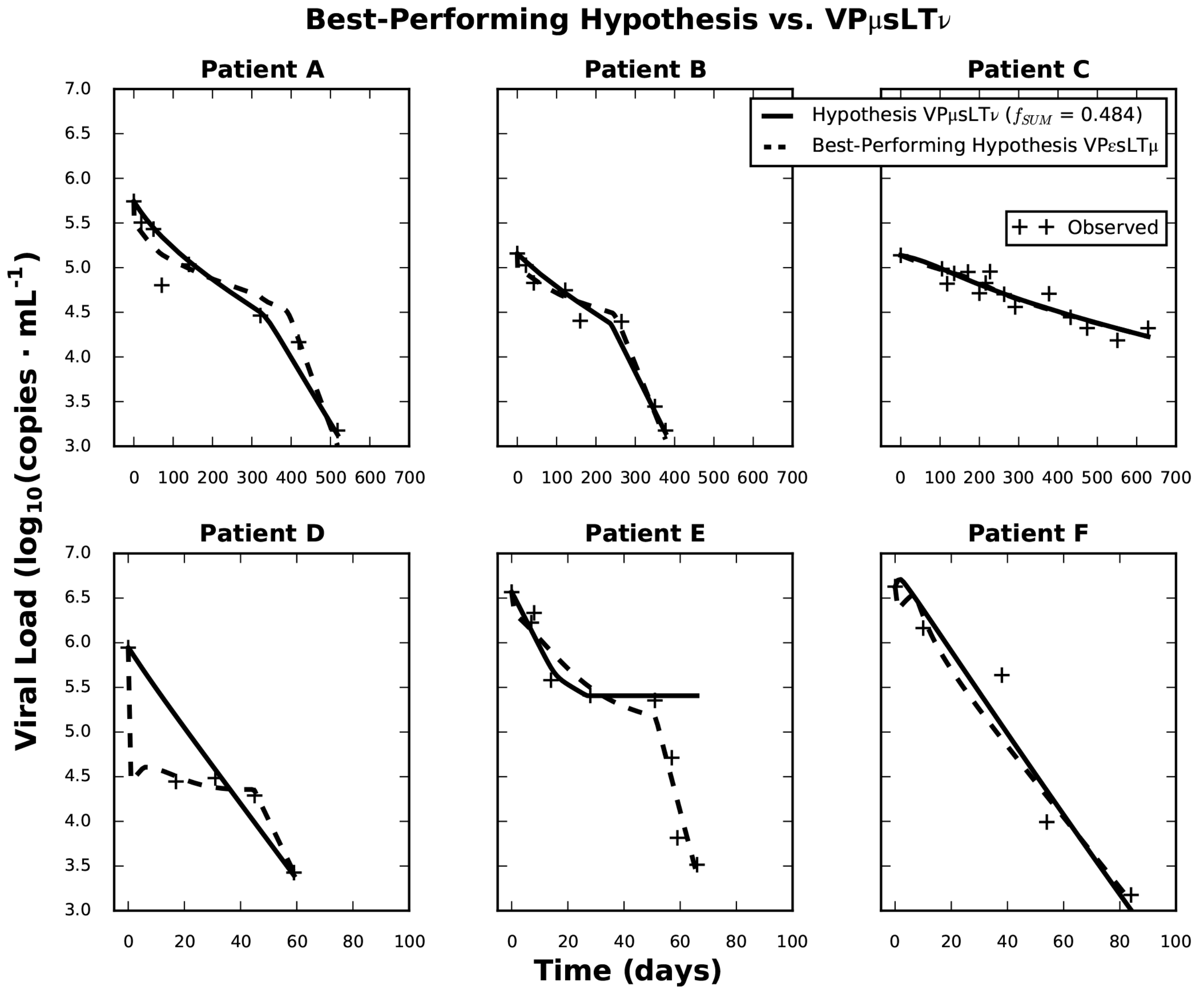
Best-Performing Hypothesis vs. VPμsLTν
Patient A
Patient B
Patient C
Patient D
Patient E
Patient F
Hypothesis VPμsLTν (f_{SUM} = 0.484)
Best-Performing Hypothesis VPεsLTμ
Observed
Viral Load ($\log_{10}$(copies · mL^{-1}))
Time (days)
7.0
6.5
6.0
5.5
5.0
4.5
4.0
3.5
3.0
0 100 200 300 400 500 600 700
0 20 40 60 80 100

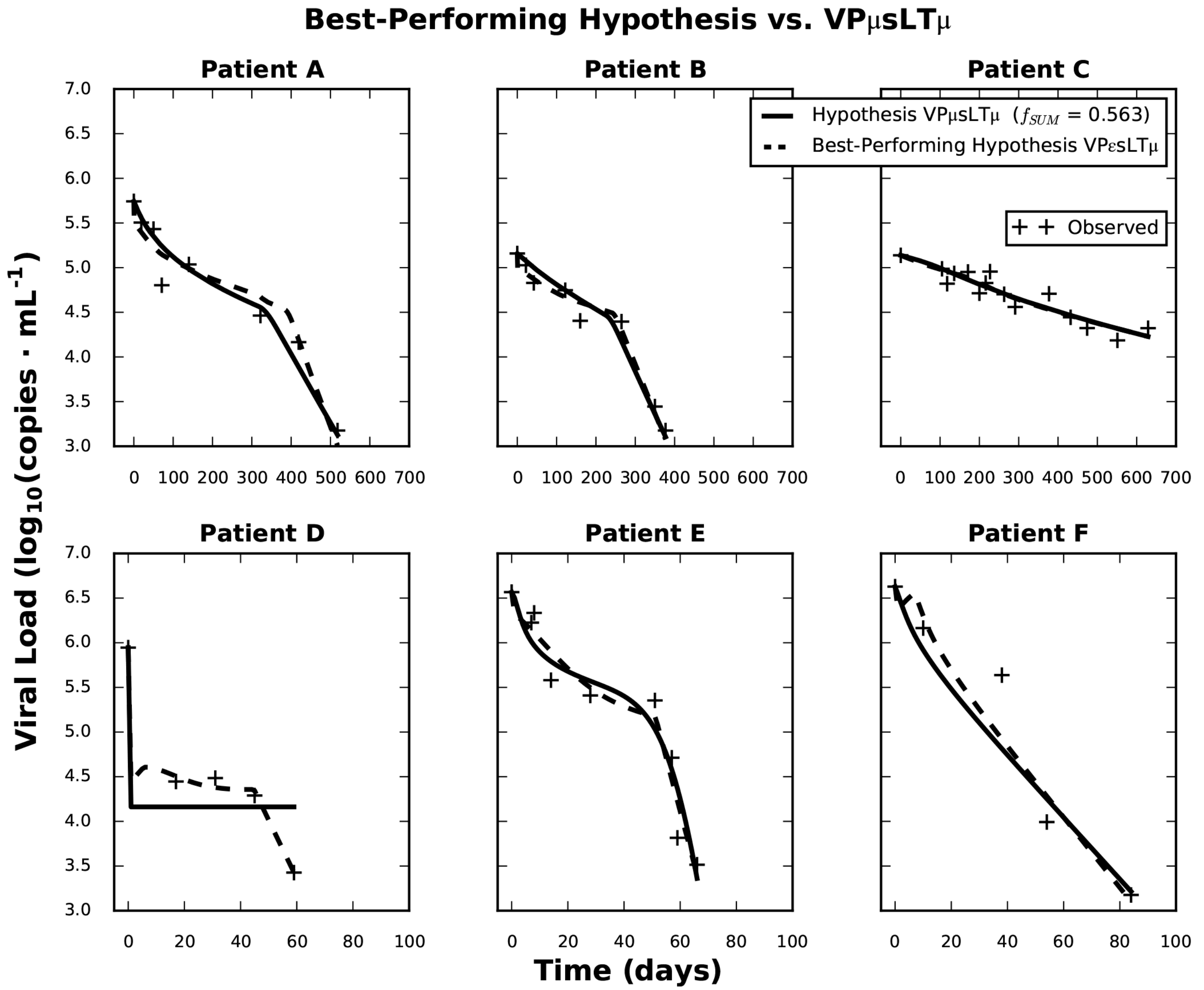

Best-Performing Hypothesis vs. VPμsLTμ
Patient A
Patient B
Patient C
Patient D
Patient E
Patient F
Hypothesis VPμsLTμ (f_{SUM} = 0.563)
Best-Performing Hypothesis VPεsLTμ
Observed
Viral Load ($\log_{10}$(copies · mL^{-1}))
Time (days)

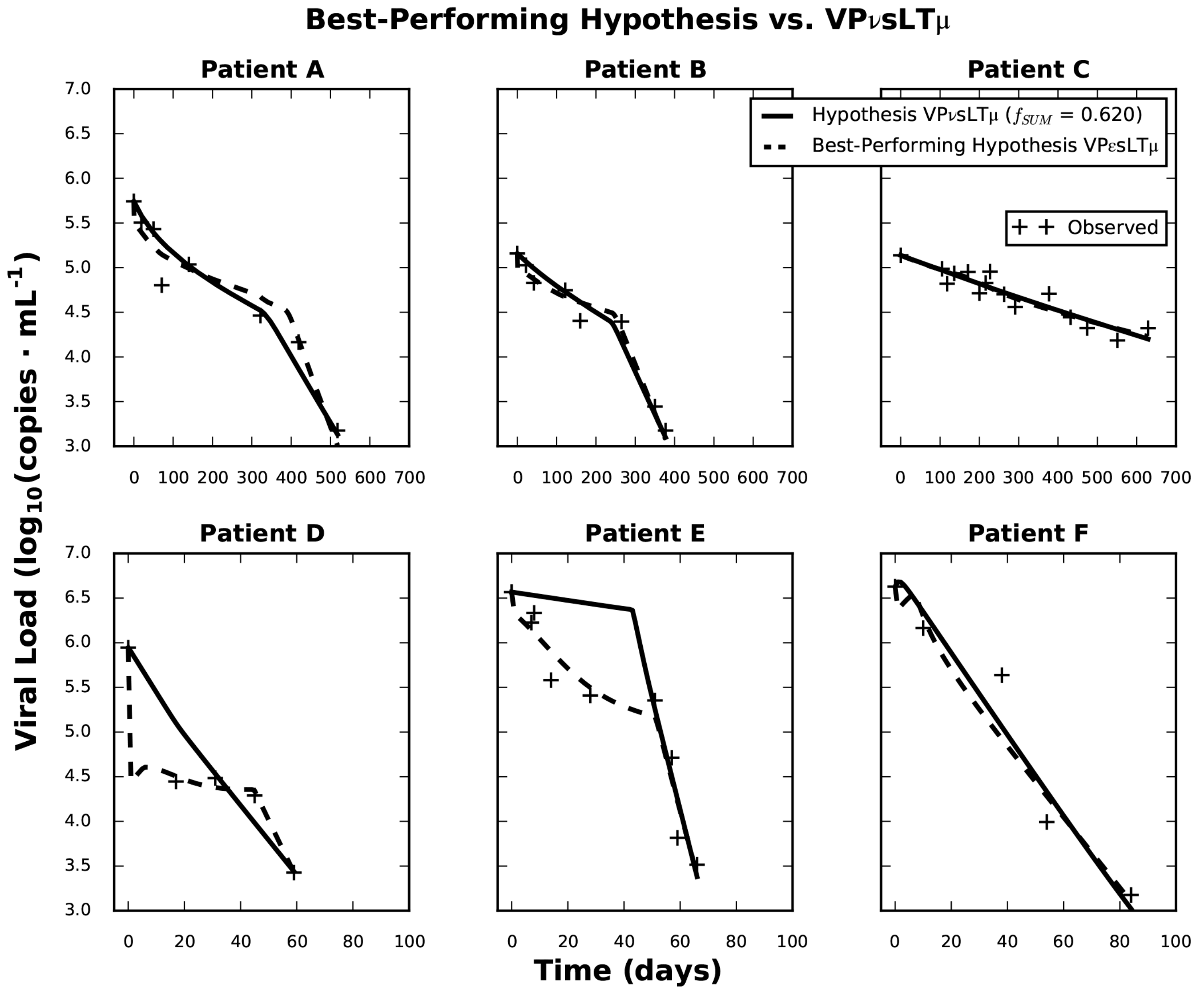

Best-Performing Hypothesis vs. VPνsLTμ
Patient A
Patient B
Patient C
Patient D
Patient E
Patient F
Hypothesis VPνsLTμ (f_{SUM} = 0.620)
Best-Performing Hypothesis VPεsLTμ
Observed
Viral Load ($\log_{10}$(copies · mL^{-1}))
Time (days)

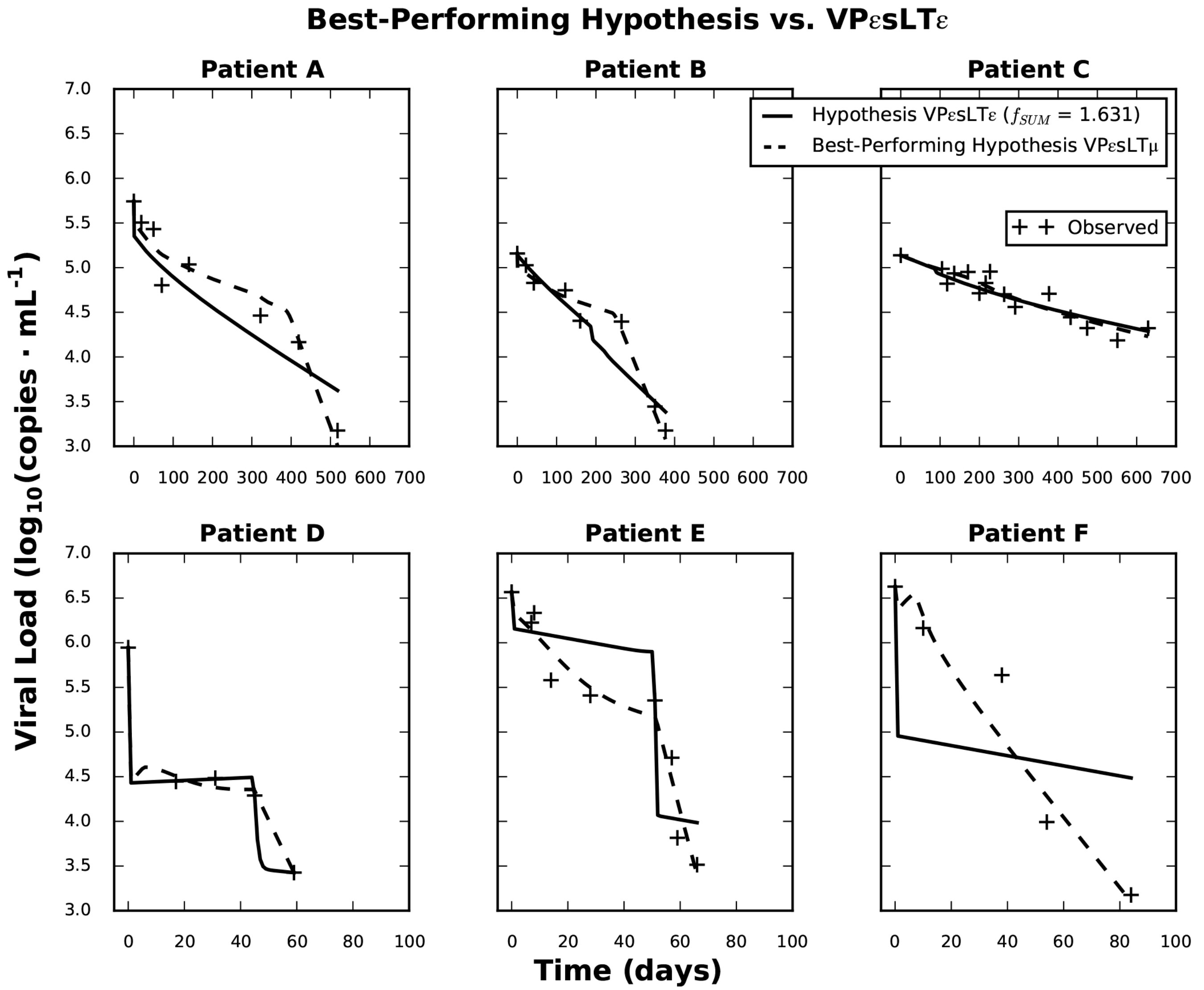

Best-Performing Hypothesis vs. VPεsLTε
Patient A
Patient B
Patient C
Patient D
Patient E
Patient F
Hypothesis VPεsLTε (f_{SUM} = 1.631)
Best-Performing Hypothesis VPεsLTμ
Observed
Viral Load ($\log_{10}$(copies · mL^{-1}))
Time (days)
7.0
6.5
6.0
5.5
5.0
4.5
4.0
3.5
3.0
0
100
200
300
400
500
600
700
20
40
60
80

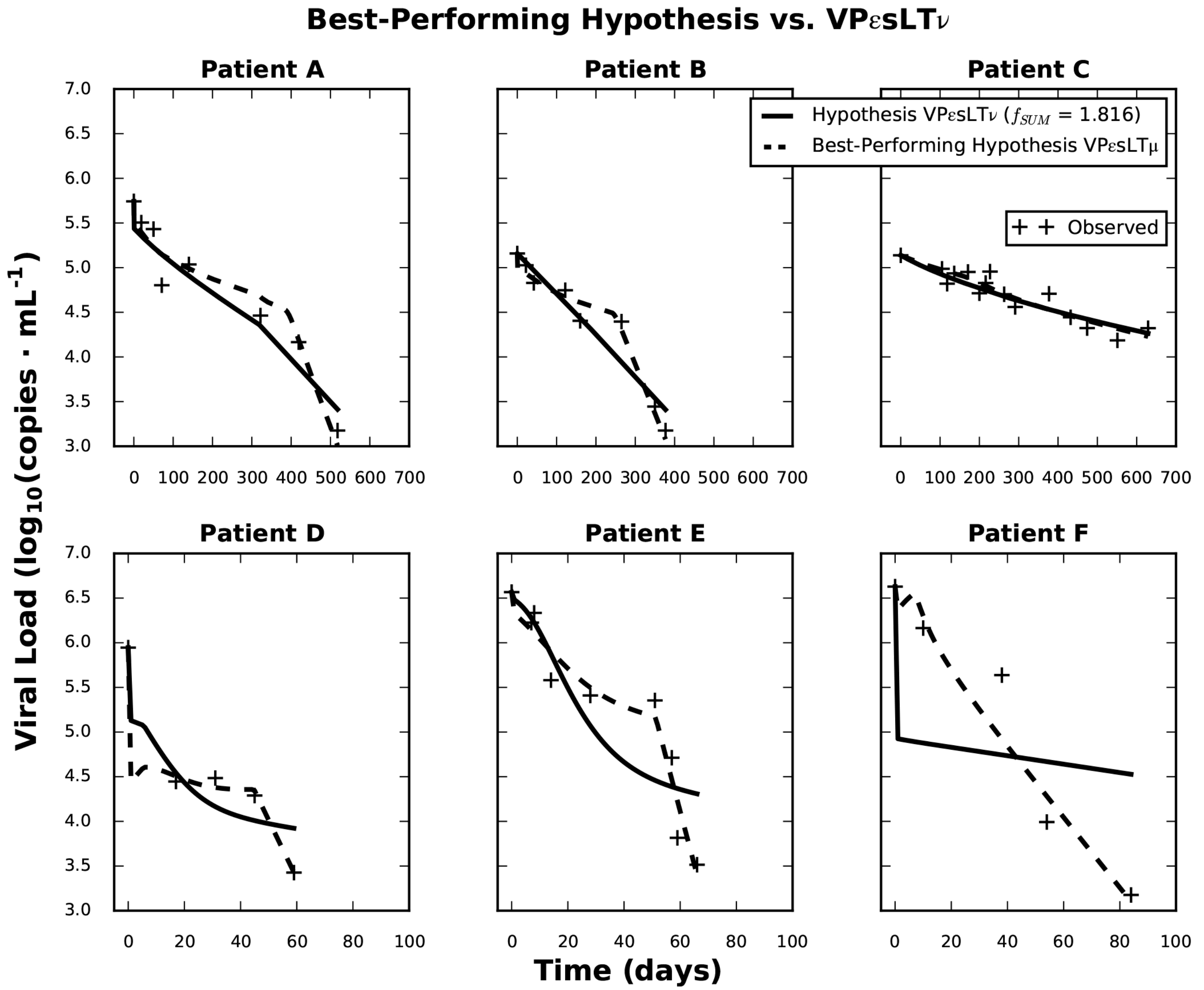
Best-Performing Hypothesis vs. VPεsLTν
Patient A
Patient B
Patient C
Patient D
Patient E
Patient F
Hypothesis VPεsLTν (f_{SUM} = 1.816)
Best-Performing Hypothesis VPεsLTμ
Observed
Viral Load ($\log_{10}$(copies · mL^{-1}))
Time (days)
7.0
6.5
6.0
5.5
5.0
4.5
4.0
3.5
3.0
0 100 200 300 400 500 600 700
0 20 40 60 80 100

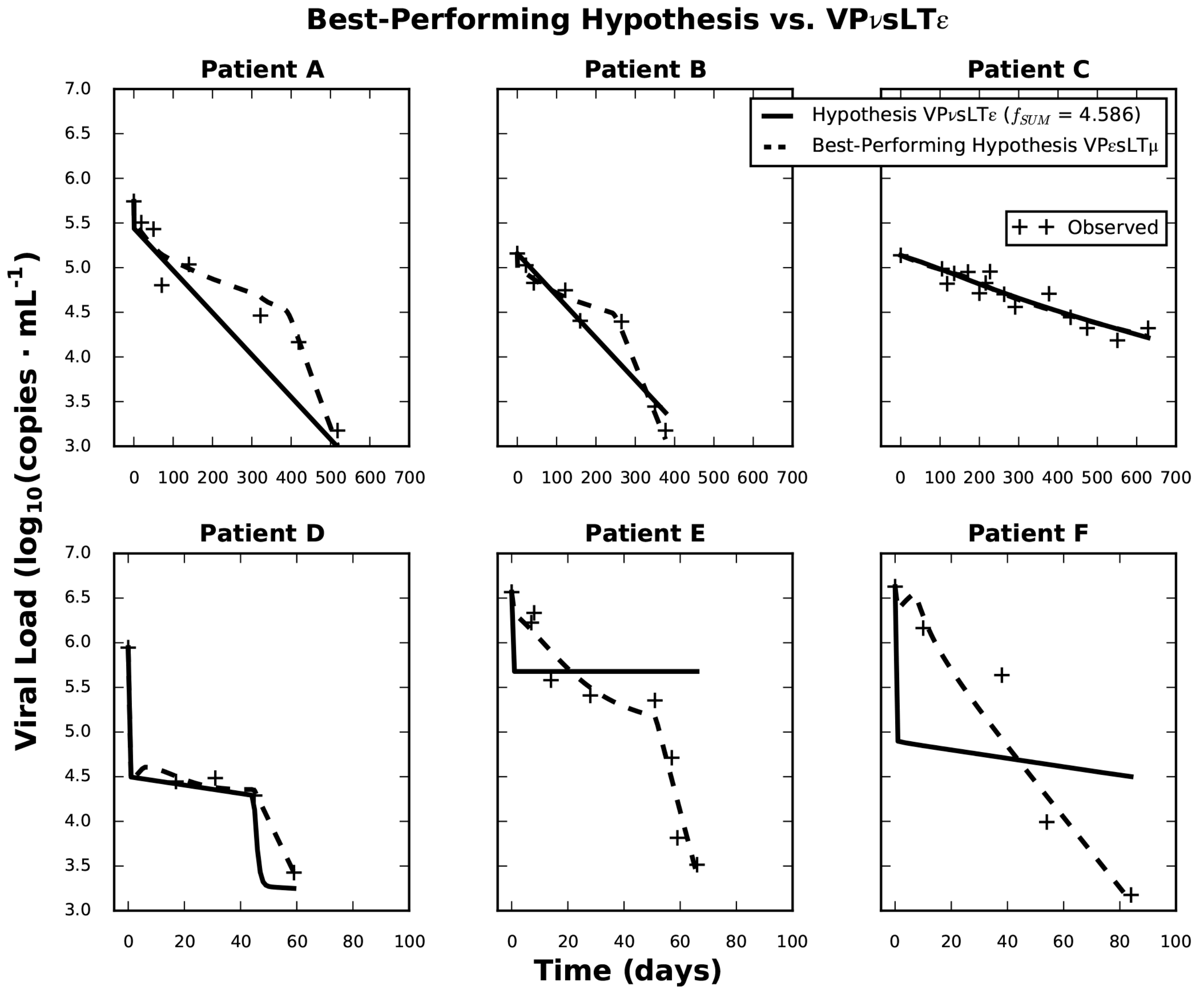

Best-Performing Hypothesis vs. VPνsLTε
Patient A
Patient B
Patient C
Patient D
Patient E
Patient F
Hypothesis VPνsLTε (f_{SUM} = 4.586)
Best-Performing Hypothesis VPεsLTμ
Observed
Viral Load ($\log_{10}$(copies · mL^{-1}))
Time (days)

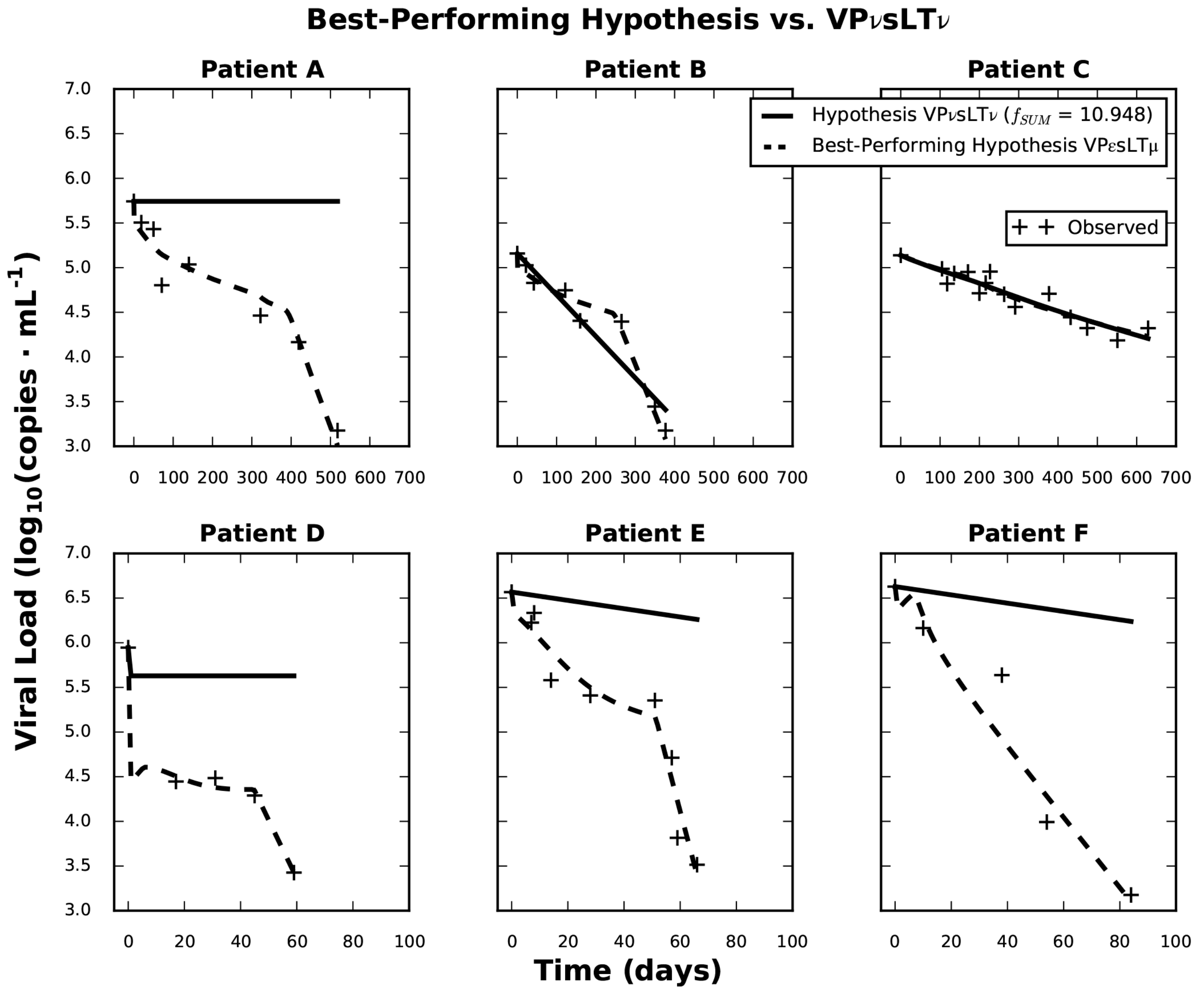

Best-Performing Hypothesis vs. VPνsLTν
Patient A
Patient B
Patient C
Patient D
Patient E
Patient F
Hypothesis VPνsLTν (f_{SUM} = 10.948)
Best-Performing Hypothesis VPεsLTμ
Observed
Viral Load ($\log_{10}$(copies · mL^{-1}))
Time (days)
7.0
6.5
6.0
5.5
5.0
4.5
4.0
3.5
3.0
0
100
200
300
400
500
600
700
20
40
60
80
100
