# Supplementary material for: Differential T cell response against BK virus regulatory and structural antigens: A viral dynamics modelling approach
Source: PLoS Comput Biol. 2018 May 10;14(5):e1005998. doi: 10.1371/journal.pcbi.1005998 (PMC5944912; doi:10.1371/journal.pcbi.1005998)
Supplement: S3 Fig — Note that for k only k = 10.7 was plotted, as k = 0.107 is not biologically meaningful. (PDF) [file pcbi.1005998.s007.pdf]

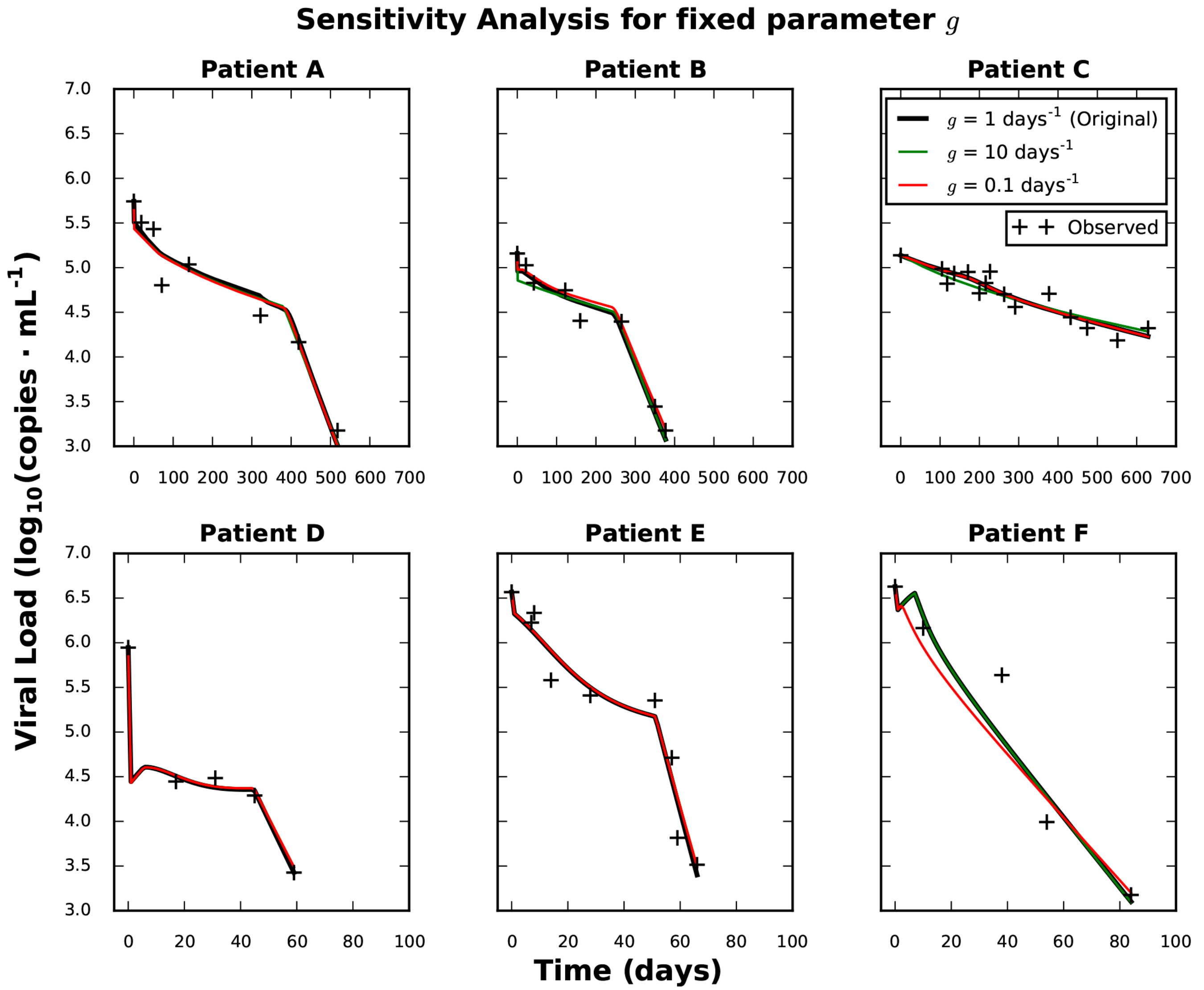
Sensitivity Analysis for fixed parameter g
Patient A
Patient B
Patient C
Patient D
Patient E
Patient F
g = 1 days-1 (Original)
g = 10 days-1
g = 0.1 days-1
Observed
Viral Load (log10(copies · mL-1))
Time (days)
7.0
6.5
6.0
5.5
5.0
4.5
4.0
3.5
3.0
0 100 200 300 400 500 600 700
0 20 40 60 80 100

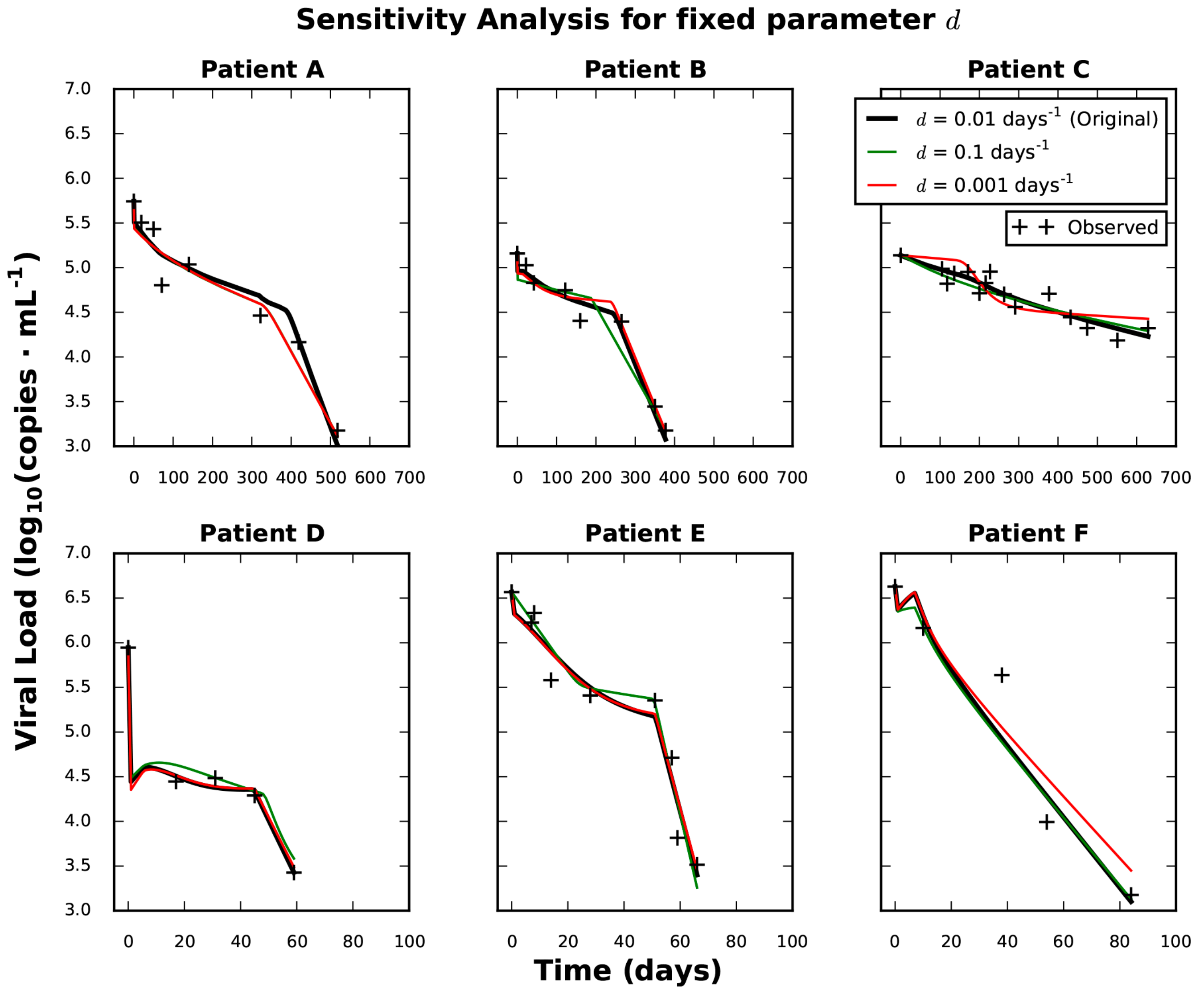
Sensitivity Analysis for fixed parameter d
Patient A
Patient B
Patient C
Patient D
Patient E
Patient F
d = 0.01 days^{-1} (Original)
d = 0.1 days^{-1}
d = 0.001 days^{-1}
Observed
Viral Load ($\log_{10}$(copies · mL^{-1}))
Time (days)

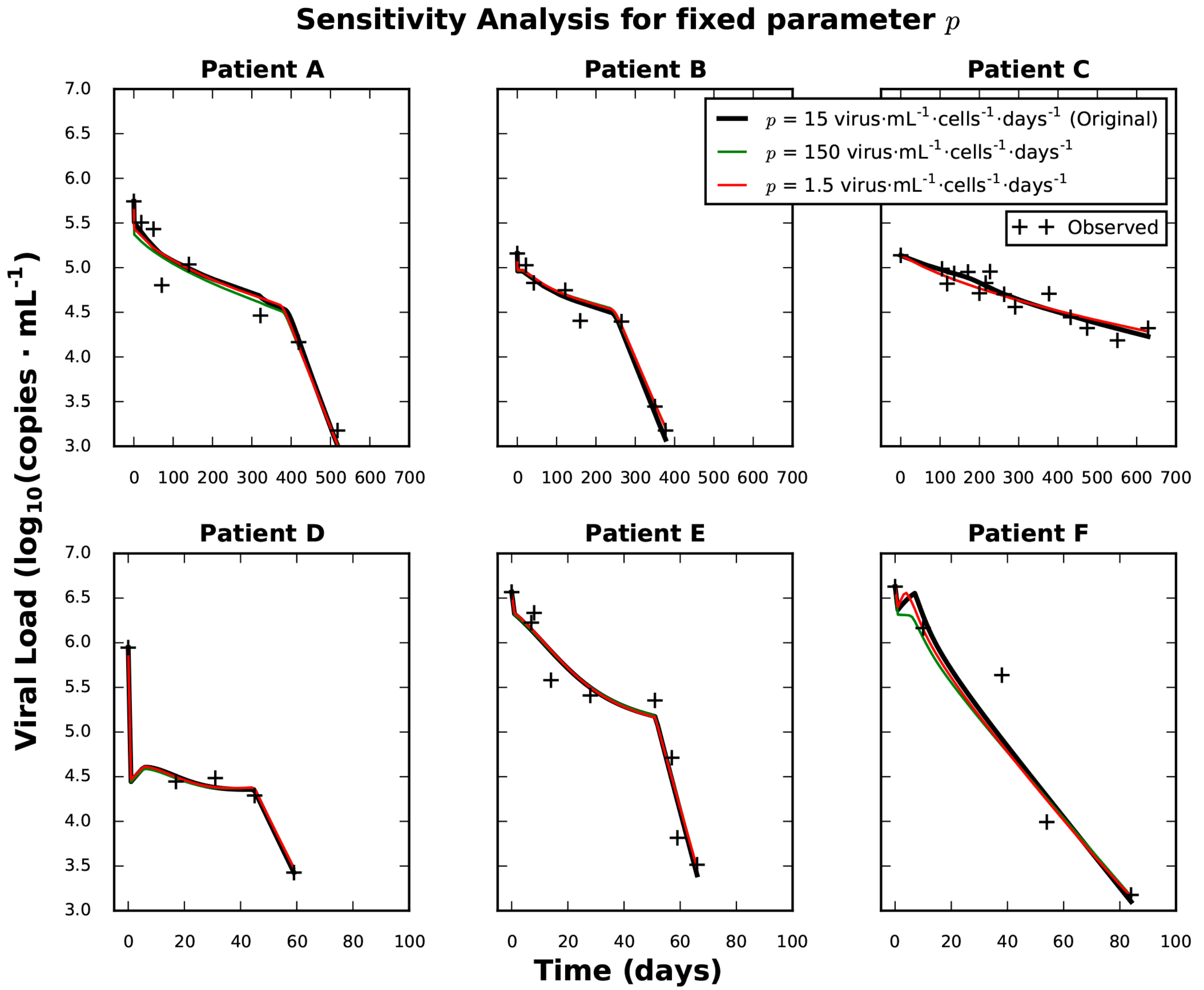

Sensitivity Analysis for fixed parameter p
Patient A
Patient B
Patient C
Patient D
Patient E
Patient F
p = 15 virus·mL^{-1}·cells^{-1}·days^{-1} (Original)
p = 150 virus·mL^{-1}·cells^{-1}·days^{-1}
p = 1.5 virus·mL^{-1}·cells^{-1}·days^{-1}
+ + Observed
Viral Load ($\log_{10}$(copies · mL^{-1}))
Time (days)
7.0
6.5
6.0
5.5
5.0
4.5
4.0
3.5
3.0
0
100
200
300
400
500
600
700
20
40
60
80
100

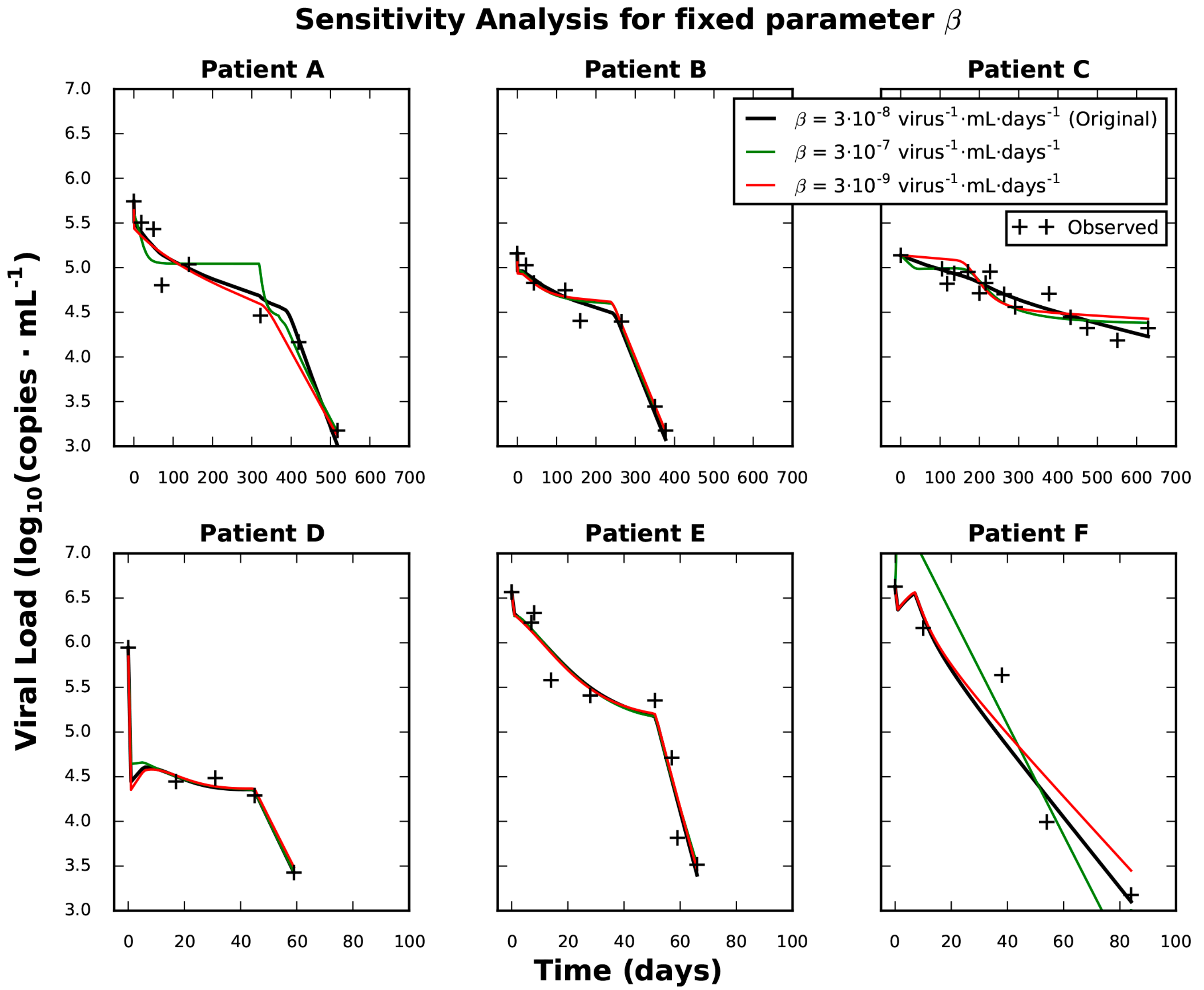
Sensitivity Analysis for fixed parameter β
Patient A
Patient B
Patient C
Patient D
Patient E
Patient F
β = 3·10^-8 virus^-1·mL·days^-1 (Original)
β = 3·10^-7 virus^-1·mL·days^-1
β = 3·10^-9 virus^-1·mL·days^-1
+ Observed
Viral Load (log10(copies · mL^-1))
Time (days)

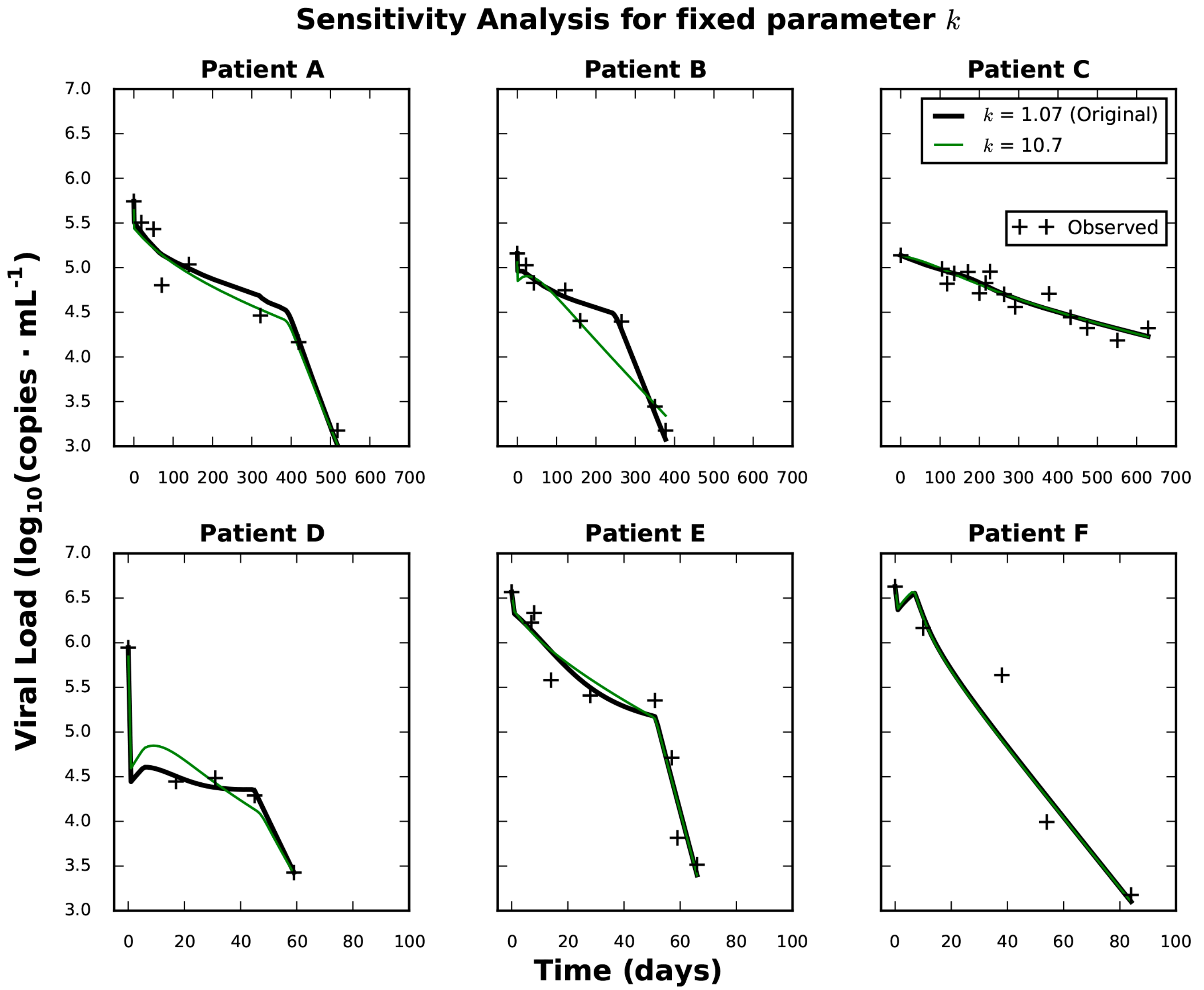

Sensitivity Analysis for fixed parameter k
Patient A
Patient B
Patient C
Patient D
Patient E
Patient F
k = 1.07 (Original)
k = 10.7
Observed
Viral Load ($\log_{10}$(copies · mL^{-1}))
Time (days)
